# Efficacy and Safety of a Tailored Dosing Strategy with High-Dose IncobotulinumtoxinA at Flexible Injection Intervals for Cervical Dystonia: An Open-Label, Uncontrolled, Single-Arm Study in Japan

**DOI:** 10.3390/neurolint18070136

**Published:** 2026-07-15

**Authors:** Akira Tamagawa, Masahiro Horiuchi, Takenori Abe, Shinichi Matsumoto, Yohei Mukai, Kunihiko Ikeguchi, Tomoo Mano, Masahito Mihara, Kimiyoshi Arimura, Kotaro Asanuma, Kanako Kurihara, Sonoko Misawa, Ryosuke Miyamoto, Noriko Nishikawa, Yuzuru Sasaki, Shohei Tateishi, Yusaku Nakamura

**Affiliations:** 1 Tamagawa Clinic, Yokohama 241-0814, Japan; akira@tamagawa-clinic.com; 2 Hakone Rehabilitation Hospital Attached Yuiodawara Clinic, Odawara 250-0055, Japan; 3 Department of Neurology, Nakamura Memorial Hospital, Sapporo 060-8570, Japan; 4 Department of Neurology, Osaka Neurological Institute, Toyonaka 561-0836, Japan; dyt1mc@yahoo.co.jp; 5 Department of Neurology, National Center of Neurology and Psychiatry, Kodaira 187-8551, Japan; 6 Jichi-idai Station Brain Clinic, Shimotsuke 329-0403, Japan; 7 Department of Rehabilitation, Nara Prefectural General Medical Center, Nara 630-8054, Japan; 8 Department of Neurology, Nara Medical University, Kashihara 634-8521, Japan; 9 Department of Neurology, Kawasaki Medical School, Kurashiki 701-0192, Japan; mihara@med.kawasaki-m.ac.jp; 10 Ookatsu Neurology and Rehabilitation Hospital, Kagoshima 890-0067, Japan; 11 Yanaginobanba Takeda Clinic, Kyoto 604-8113, Japan; 12 Department of Neurology, Faculty of Medicine, Fukuoka University, Fukuoka 814-0180, Japan; 13 Department of Neurology, Graduate School of Medicine, Chiba University, Chiba 260-8670, Japan; 14 Department of Neurology and Neurological Science, Institute of Science Tokyo, Bunkyo-ku 113-8519, Japan; 15 Department of Neurology, Tokushima University Graduate School of Biomedical Sciences, Tokushima 770-8503, Japan; ryom@tokushima-u.ac.jp; 16 Department of Neurology, Juntendo University Faculty of Medicine, Bunkyo-ku 113-8421, Japan; 17 Clinical Development Department, Teijin Pharma Limited, Chiyoda-ku 100-8585, Japan; 18 Clinical Development Control Department, Teijin Pharma Limited, Chiyoda-ku 100-8585, Japan; 19 Department of Neurology, Rinku General Medical Center, Izumisano 598-8577, Japan

**Keywords:** botulinum toxin, tardive dystonia, efficacy, incobotulinumtoxinA, Japan, tailored dosing, safety, cervical dystonia, tardive dyskinesia

## Abstract

Background: This prospective, multicenter, open-label, single-arm study (jRCT2031230690) evaluated the efficacy and safety of a tailored dosing strategy of incobotulinumtoxinA, including high doses (up to 500 U) and flexible injection intervals (as short as 6 weeks), in Japanese patients with cervical dystonia (CD). Methods: Of 30 enrolled patients, Group A included 27 patients with idiopathic CD for the primary evaluation of efficacy and safety, whereas Group B included 3 patients with tardive dyskinesia (cervical) or tardive CD for exploratory safety assessment. Patients received up to seven injection cycles of incobotulinumtoxinA (120–500 U) over 48 weeks, with minimum 6-week intervals. The primary endpoint, evaluated in Group A, was the change in Toronto Western Spasmodic Torticollis Rating Scale (TWSTRS) total score from baseline to Week 4 after the first injection. Results: Using a mixed model for repeated measures, the least squares mean ± standard error of the change was −11.0 ± 1.77 (95% confidence interval: −14.6, −7.3). The primary efficacy endpoint was achieved in Group A. Due to the small sample size (*n* = 3), efficacy in Group B was evaluated only for exploratory purposes, although safety findings were broadly consistent with those in Group A. The overall safety profile was consistent with previous studies. Across the study, the most common related adverse events were dysphagia (33.3%) and muscular weakness (22.2%) in Group A and dysphagia (33.3%) in Group B. All cases of dysphagia were mild to moderate in severity and transient, with no apparent dose- or injection interval-related trend observed. Conclusions: IncobotulinumtoxinA was associated with improvements in symptoms and manageable safety profile at high doses and flexible injection intervals in Japanese patients with CD. While these findings suggest a potential treatment option for individualized dose optimization, the absence of a control group and the exploratory nature of the assessment in Group B necessitate cautious interpretation.

## 1. Introduction

Botulinum neurotoxins, produced by Clostridia, cause botulism by paralyzing motor axon nerve terminals, leading to transient muscle weakness [1]. These neurotoxins penetrate nerve terminals and cleave proteins essential for the release of vesicular acetylcholine. Specifically, botulinum neurotoxin type A, which is used in human therapy, targets and cleaves the protein SNAP-25 (synaptosomal-associated protein, 25 kDa) [2,3].

Cervical dystonia (CD), also referred to as spasmodic torticollis, is a chronic condition characterized by involuntary contractions of the neck muscles, resulting in abnormal, often repetitive movements, postures, or both [4,5]. It is a common and lifelong focal dystonia wherein remissions are rare after the first year [6]. Patients with CD frequently exhibit a spectrum of symptoms, encompassing both motor and nonmotor manifestations. Motor symptoms involve sustained or intermittent contractions of the neck muscle, resulting in abnormal head postures and tremors [7]. Nonmotor symptoms include psychiatric disorders, cognitive impairment, sleep disturbances, and sensory abnormalities, all of which significantly affect quality of life (QoL) and contribute to disability in individuals with CD [8].

CD is the most prevalent form of adult-onset focal dystonia, with prevalence estimates ranging from 57 per million in Europe, to as high as 280 per million in the United States (US) [4,9]. The onset typically occurs in the fourth or fifth decade of life and is more common in women than men, with a female-to-male ratio of approximately 2:1 [10].

The first-line treatment for CD widely accepted in many countries is botulinum toxin [4,9,11], and, in Japan, onabotulinumtoxinA was the first formulation available for clinical use [12]. Reports suggest that patient satisfaction with botulinum toxin therapy varies, reflecting heterogeneity in disease severity, muscle involvement, and individual treatment requirements [13]. Notably, the most common reason for suboptimal treatment response has been reported to be inadequate dosing [13].

IncobotulinumtoxinA is a highly purified botulinum neurotoxin type A product, formulated to include solely the 150 kDa neurotoxin as the active component with unnecessary clostridial proteins removed, and exhibiting low immunogenicity [14]. The efficacy and safety of incobotulinumtoxinA have been validated in several international clinical studies [15,16,17,18,19,20,21,22,23]. IncobotulinumtoxinA is approved for the treatment of CD in several countries, including those in Europe, and the US [24,25]. However, it has not yet received approval for CD in Japan [26]. The maximum approved doses of onabotulinumtoxinA and incobotulinumtoxinA for CD vary by country or region, with country-specific limits of 240 U for onabotulinumtoxinA in Japan, 300 U for both onabotulinumtoxinA and incobotulinumtoxinA in Europe, and 300 U for onabotulinumtoxinA or 240 U for incobotulinumtoxinA in the US. Despite this, previous studies have reported administered doses ranging from 40 U to 860 U, with a mean ± standard deviation (SD) of 262.6 ± 141.6 U, indicating that doses exceeding 300 U are sometimes used in clinical practice [27]. Given the clinical need for individualized dose optimization to address diverse treatment requirements, and the specific lack of evidence for those requiring doses higher than 300 U, generating clinical evidence for a tailored dosing strategy that permits such high-dose administration is crucial.

In addition to dose optimization, there is a growing need for shorter injection intervals among patients with CD. Previous studies have confirmed that it is common for patients with CD to experience symptom re-emergence between botulinum injections (88%), with the time from injection to symptom re-emergence reported as 73.6 days (approximately 10.5 weeks). As the treatment effect wanes, symptom severity increases, impacting QoL [28]. To address these clinical gaps, evaluating the potential for more flexible injection intervals is necessary. Therefore, establishing the clinical utility of such an approach, particularly by exploring cycles as short as 6 weeks to accommodate patients with early symptom re-emergence, is critical as a key component of a tailored dosing strategy.

Accordingly, this prospective, multicenter, open-label, uncontrolled, single-arm clinical study is the first to evaluate the efficacy and safety of incobotulinumtoxinA for the treatment of CD in Japan. It was conducted as part of the clinical development program, following regulatory agreement, to support the clinical evaluation of incobotulinumtoxinA for the indication of CD. The study primarily focused on patients with idiopathic CD, while also including a small cohort of patients with tardive symptoms for exploratory safety assessment. The study focused on a tailored dosing strategy, incorporating high doses of up to 500 U and flexible injection intervals as short as 6 weeks, for both botulinum toxin-naïve patients and those with a history of previous botulinum toxin treatment.

## 2. Materials and Methods

### 2.1. Study Design

**Figure 1:**
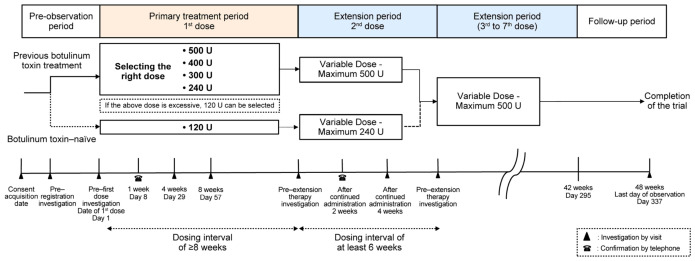
Study design.

This was a prospective, multicenter, open-label, uncontrolled, single-arm study (Japanese Registry of Clinical Trials: jRCT 2031230690) conducted as part of the clinical development program following regulatory agreement (Figure 1). The study was registered on March 12, 2024, and conducted between 10 April 2024, and 9 July 2025 (48 weeks after the first dose of the study drug in the last patient), at 15 medical institutions. The study population was categorized into two groups based on clinical characteristics. Group A included patients diagnosed with idiopathic CD, and Group B comprised patients with tardive dyskinesia (cervical) or tardive CD. Group A was established to evaluate the efficacy and safety of incobotulinumtoxinA in Japanese patients with idiopathic CD, whereas Group B was exclusively intended for exploratory safety assessment in patients with tardive dyskinesia or tardive CD.

### 2.2. Selection Criteria

#### 2.2.1. Inclusion Criteria

Eligible participants were male or female patients aged between 18 and 75 years. All patients were required to have a clinical diagnosis of CD, a Toronto Western Spasmodic Torticollis Rating Scale (TWSTRS) total score of ≥20 points, a TWSTRS severity score of ≥10 points, a TWSTRS disability score of ≥3 points, and a TWSTRS pain score of ≥1 point. Patients with previous botulinum toxin treatment for CD were eligible if at least 10 weeks had passed since their last injection at the time of screening, if the effect of the previous treatment had diminished and further treatment was deemed necessary by the investigator, and if the previous treatment had been effective in the investigator’s judgment. Patients clinically diagnosed with tardive dyskinesia (cervical) or tardive dystonia (cervical) were also eligible (applicable to Group B only).

#### 2.2.2. Exclusion Criteria

Patients were excluded if they had traumatic or congenital dystonia, presenting only with symptoms of forward flexion, or other than forward flexion, with a TWSTRS severity score of ≥2 points for forward flexion. Additional exclusion criteria included a history of deep brain stimulation, intrathecal baclofen therapy, or surgical interventions for CD (e.g., myotomy and selective peripheral nerve blockade); significantly limited passive range of motion in the neck due to contractures or deformities; generalized neuromuscular junction disorders (e.g., myasthenia gravis and Lambert-Eaton syndrome) or amyotrophic lateral sclerosis; botulinum toxin treatment for indications other than CD less than 16 weeks prior to screening; and a history of primary or secondary non-responsiveness to previous botulinum toxin treatment as judged by the investigator. Patients who had received anticoagulants (e.g., heparin and warfarin) less than 1 week prior to screening were excluded, although antiplatelet drugs were permitted. Patients with infections at the intended injection site, systemic infections that would make cervical muscle injection unsafe, or hypersensitivity to botulinum toxin (any serotype) or to excipients of the study drug (e.g., human serum albumin and purified sucrose) were also excluded. Patients with tardive dyskinesia (cervical) or tardive dystonia (cervical) and those presenting only with symptoms of backward extension or with a TWSTRS severity score for backward extension of ≥2 points at screening were excluded (applicable to Group A only).

### 2.3. Treatment

Botulinum toxin-naïve patients received a fixed initial dose of 120 U, whereas patients with previous botulinum toxin treatment received a physician-selected dose of 240 U, 300 U, 400 U, or 500 U based on the patients’ symptoms. For patients in whom 240 U was deemed excessive, a reduced dose of 120 U was chosen and administered instead. The second dose was administered as a variable dose, at least 8 weeks after the first dose, contingent upon meeting re-administration criteria, with botulinum toxin-naïve patients receiving up to 240 U and patients with previous botulinum toxin treatment receiving up to 500 U. The third and subsequent doses were administered at least 6 weeks after the previous dose, based on re-administration criteria, with a maximum dose of 500 U for all patients.

### 2.4. Endpoints

#### 2.4.1. Efficacy

The primary endpoint was the change in the TWSTRS total score from baseline to Week 4 following the first dose (assessed as effective when the upper limit of the 95% confidence interval [CI] was below the threshold of −3.0). This threshold was defined based on the maximum treatment effect with placebo at 4 weeks after the first injection, as assumed from the results of the rimabotulinumtoxinB Phase 3 trial in Japan [29], which remains the only large placebo-controlled trial in Japanese patients with CD using TWSTRS as the primary outcome. This threshold was also considered to represent the minimum clinically meaningful effect, based on the results of an overseas Phase 3 clinical trial of incobotulinumtoxinA [16]. TWSTRS is a composite scale developed by Consky and Lang [30,31], which measures the severity of CD [32,33] using three subscales: the severity subscale, which assesses physical findings; the disability subscale, which evaluates the impact on work and activities of daily living (ADL); and the pain subscale, which measures head and neck pain. The total score is the sum of the three subscale scores.

#### 2.4.2. Secondary Endpoints

Secondary endpoints included reduction in the TWSTRS total score (excluding the change from baseline to Week 4 after the first injection); TWSTRS severity, disability, and pain scores; Cervical Dystonia Impact Profile-58 (CDIP-58), assessed on a 5-point scale (1 “not at all” to 5 “very/always”) across 58 items covering head and neck symptoms, pain and discomfort, upper extremity movement, gait, sleeping, irritability, mood, and psychosocial functioning [34,35]; and Patient Evaluation of Global Response (PEGR), assessed on a 9-point scale (−4 “markedly worsened” to +4 “completely absent symptom”), measuring patient-perceived changes (worsening or amelioration) in CD symptoms [36]. Use of the CDIP-58, authored by Jeremy Hobart et al., was under license from TransformMS CIC. Subscale D of the Modified Tsui Scale [37] (Head Tremor Assessment) assessed head tremor severity and duration, with scores calculated by multiplying both parameters, yielding a total of 1, 2, or 4 points. If the patient had no tremor, the score was recorded as 0 and included in the calculation. The type of CD was assessed and determined by the investigator based on the patient’s CD symptoms.

Following administration, additional analyses were conducted to evaluate changes in the TWSTRS severity score by target muscle application site (e.g., sternocleidomastoid and trapezius) and individual severity score items such as rotation, lateral flexion, and anterior–posterior flexion. The location of the target muscles for injection was identified using electromyography, ultrasound, or anatomical landmarks. Furthermore, outcomes from subscale D of the Modified Tsui Scale (Head Tremor Assessment) were analyzed specifically in patients presenting with tremor symptoms. Safety was evaluated through the monitoring and documentation of adverse events (AEs) throughout the study period.

### 2.5. Statistical Analysis

Statistical analyses were primarily performed for Group A. For Group B, formal inferential statistical analysis of efficacy was not conducted due to the limited sample size (*n* = 3), and results were summarized descriptively for exploratory purposes.

#### 2.5.1. Primary Efficacy Endpoints

The primary endpoint was the change in the TWSTRS total score from baseline to Week 4. For the primary analysis, a mixed model for repeated measures was employed, with the change in the TWSTRS total score from baseline as the objective variable, baseline TWSTRS total score as the explanatory variable, and time point of examination as a fixed effect. Data from pre-administration assessments through 8 weeks post-administration were used for model estimation. Least squares mean (LSM) and 95% CI were calculated for each time point. Missing data were not imputed.

#### 2.5.2. Secondary Efficacy Endpoints

TWSTRS total score, along with severity, disability, and pain scores, were calculated for both measured values and changes from baseline at each injection cycle using descriptive statistics. Missing data were primarily handled using an observed case (OC) analysis. In addition, analyses using a baseline observation carried forward approach (BOCF) were conducted as a conservative sensitivity analysis to assess the robustness of the results. Based on the low level of missing data observed in the previous overseas study [16], we anticipated that missingness would also be minimal in this trial. In practice, missing data in this study were indeed minimal (3.7% at Week 8), which supported the appropriateness of using simple and transparent approaches. CDIP-58, PEGR, and subscale D of the Modified Tsui Scale were calculated using categorical tabulation and descriptive statistics at each injection cycle. For missing values, an observed case analysis was performed.

#### 2.5.3. Safety Endpoint

For AEs and related AEs, the number of patients experiencing events and the incidence were summarized by severity at the time of onset. Serious AEs were defined as events resulting in death, life-threatening situations, requiring hospitalization or extension of hospitalization for treatment, causing persistent or significant disability or dysfunction, congenital anomalies, or other medically important conditions. Additionally, the names of AEs documented by the physician-in-charge on the case report form were replaced by lower-layer terms according to the Medical Dictionary for Regulatory Activities (MedDRA) and tabulated using system organ class and preferred term. AEs and related AEs were coded using MedDRA version 27.1, and statistical analysis was performed using SAS version 9.4.

#### 2.5.4. Sample Size Determination

Group A was established to evaluate the efficacy and safety of incobotulinumtoxinA in Japanese patients with idiopathic CD, using the same population as in a previous trial [16]. The expected value for the anticipated treatment effect of incobotulinumtoxinA was set at −9.9 points, based on the mean change in TWSTRS total score 4 weeks after the first 120 U dose of incobotulinumtoxinA in the overseas Phase 3 trial [16]. Using the abovementioned threshold (−3.0 points) and expected value (−9.9 points), with the SD for change set at 10.35 points, a one-sided significance level of 2.5%, and power of 80%, the required sample size based on a one-sample *t*-test was 20 patients. Therefore, considering the possibility of trial termination, the target sample size was set to 22 patients. Regarding the sample size setting for Group B, the 2020 patient survey by the Ministry of Health, Labour and Welfare in Japan reported no cases of drug-induced dystonia [38]. Based on this finding, the sample size for Group B was not predefined, and the number of patients was set as high as possible for safety assessment, taking feasibility into consideration.

## 3. Results

### 3.1. Patient Disposition

A total of 30 patients were enrolled and received the study drug. Group A (*n* = 27), the primary evaluation group, included patients with idiopathic CD. Group B (*n* = 3), the exploratory cohort, comprised patients with tardive dyskinesia (cervical) or tardive CD.

**Figure 2:**
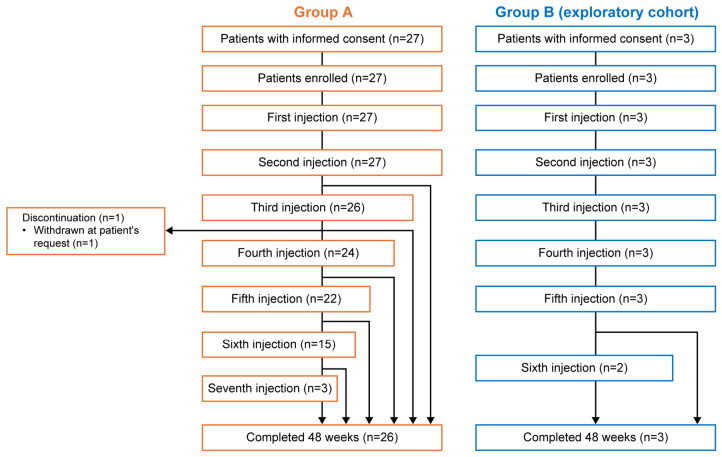
Patient disposition (until 48 weeks).

By the 48-week mark, 29 patients had received repeated injections. The number of patients for each injection cycle is summarized in Figure 2. One patient in Group A discontinued treatment after the third injection and did not proceed to receive the fourth injection. The reason for discontinuation was “withdrawn at patient’s request.” All 30 patients were included in both the full analysis set (FAS) and safety population.

### 3.2. Demographic and Baseline Characteristics

All of the 30 patients included in the FAS were Japanese (Asian race), of whom 20 (66.7%) were male and 10 (33.3%) were female (Table 1). The primary focus of this study was Group A (*n* = 27), comprising patients with idiopathic CD, whereas Group B (*n* = 3) was included for safety assessment.

In Group A, the mean duration of CD was 92.58 ± 123.9 months. Of these 27 patients, 7 (25.9%) were botulinum toxin-naïve and 20 (74.1%) had previously received botulinum toxin treatment. The mean baseline TWSTRS total score in Group A was 47.07 ± 7.45 for botulinum toxin-naïve patients and 45.95 ± 11.32 for those with previous treatment (overall mean: 46.24 ± 10.33). Regarding disease severity, 8 patients (29.6%) had moderate CD (TWSTRS severity score 16–21) and 19 (70.4%) were classified as having severe CD (TWSTRS severity score 22–35).

Group B, the exploratory cohort, consisted of 3 patients with a mean CD duration of 111.17 ± 31.0 months. All 3 patients (100%) had a history of previous botulinum toxin treatment and presented with severe CD at baseline (mean TWSTRS total score: 45.75 ± 1.32).

### 3.3. Dosage of IncobotulinumtoxinA

For the primary evaluation group (Group A), among patients with previous botulinum toxin treatment for CD (20 patients), 1 (3.7%) received a dose of 120 U, 2 (7.4%) received 240 U, 3 (11.1%) received 300 U, 6 (22.2%) received 400 U, and 8 (29.6%) received 500.0 U at the first injection. In the exploratory cohort (Group B), 1 patient each with previous botulinum toxin treatment received doses of 120 U, 400 U, and 500 U. The dosage of incobotulinumtoxinA is summarized in Table 2.

### 3.4. Dosing Interval of IncobotulinumtoxinA (Group A)

In Group A, the dosing interval (mean ± SD) between the first and second injections in botulinum toxin-naïve patients and those with previous botulinum toxin treatment was 9.39 ± 1.8 and 8.72 ± 1.1 weeks, respectively (Table S1), while the interval between the second and third injections was 8.62 ± 1.8 weeks and 8.32 ± 2.7 weeks, respectively. Following the second injection, administration was confirmed at a minimum interval of 6 weeks.

### 3.5. Dose of IncobotulinumtoxinA by Target Muscle Group During the First Injection Cycle

Across the entire study population (*n* = 30), for the target muscle and dosage (mean ± SD) where an initial dose of ≥300 U was administered, the paraspinal muscle was the primary injection site (mean ± SD dose: 246.7 ± 205.3 U, *n* = 3). The splenius capitis was the next most frequently injected target muscle, receiving a dose of 133.8 ± 85.3 U (*n* = 30) (Table S2).

### 3.6. Endpoints

#### 3.6.1. TWSTRS Total Score (Group A)

In Group A (*n* = 27), the LSM ± standard error (SE) of the change in the TWSTRS total score from baseline to 4 weeks after the administration, the primary endpoint, was −11.0 ± 1.77 (95% CI: −14.6, −7.3). The upper limit of the 95% CI (−7.3) was below the prespecified threshold of efficacy (−3.0); thus, the primary endpoint was achieved.

#### 3.6.2. Reduction in the TWSTRS Total Score (Group A)

**Figure 3:**
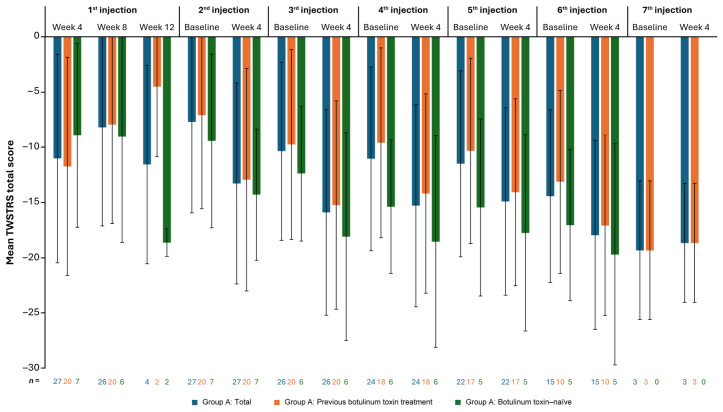
TWSTRS total score (mean ± SD) change from the 1st injection baseline at each injection cycle by treatment group in Group A. SD, standard deviation; TWSTRS, Toronto Western Spasmodic Torticollis Rating Scale.

In Group A, the reduction (change from the first injection baseline) in the TWSTRS total score (mean ± SD) observed at Week 4 (*n* = 7) and Week 8 (*n* = 6) for botulinum toxin-naïve patients was −8.93 ± 8.3 and −9.04 ± 9.6, respectively, at the first injection (Figure 3), while in patients with previous botulinum toxin treatment, it was −11.74 ± 9.9 and −7.93 ± 9.0 at Week 4 (*n* = 20) and Week 8 (*n* = 20), respectively.

At Week 12 (*n* = 4), a marked reduction in the TWSTRS total score (mean ± SD) in Group A of −11.56 ± 9.0 at the first injection was observed. The change in the TWSTRS total score from the first injection baseline by target muscle is shown in Table S3.

#### 3.6.3. Reduction in the TWSTRS Severity Score (Group A)

**Figure 4:**
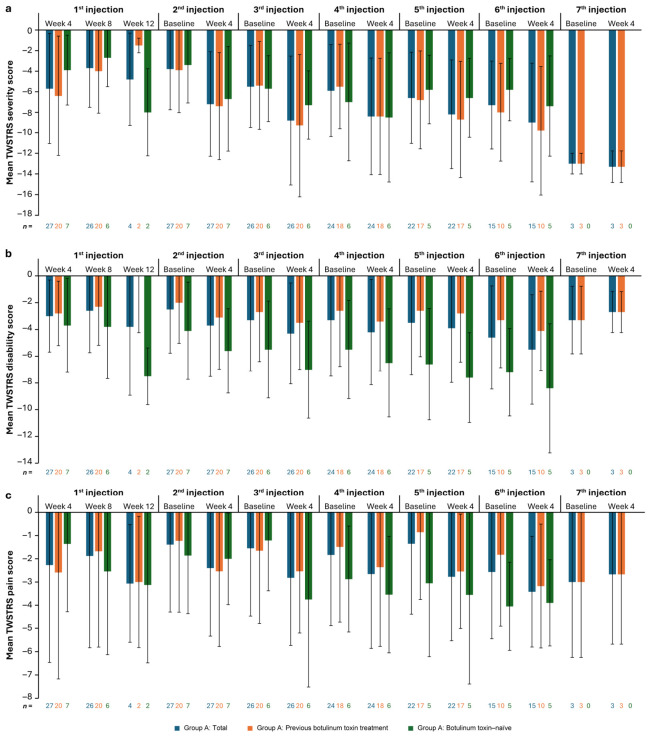
TWSTRS subscores for (**a**) severity, (**b**) disability, and (**c**) pain at each injection cycle by treatment group in Group A. TWSTRS, Toronto Western Spasmodic Torticollis Rating Scale.

In Group A, the reduction (change from the first injection baseline) in the TWSTRS severity score (mean ± SD) observed at Week 4 (*n* = 7) and Week 8 (*n* = 6) for botulinum toxin-naïve patients was −3.9 ± 3.4 and −2.7 ± 2.8, respectively, during the first injection cycle (Figure 4a), while in patients with previous botulinum toxin treatment, it was −6.4 ± 5.8 and −4.0 ± 4.1 at Week 4 (*n* = 20) and Week 8 (*n* = 20), respectively. At Week 12 (*n* = 4), the reduction in the TWSTRS severity score (mean ± SD) in Group A was −4.8 ± 4.5.

#### 3.6.4. Reduction in the TWSTRS Disability Score (Group A)

In Group A, the reduction (change from the first injection baseline) in TWSTRS disability score (mean ± SD) observed at Week 4 (*n* = 7) and Week 8 (*n* = 6) for botulinum toxin-naïve patients was −3.7 ± 3.5 and −3.8 ± 3.9, respectively, during the first injection cycle (Figure 4b), while in patients with previous botulinum toxin treatment, it was −2.8 ± 2.4 and −2.3 ± 2.9 at Week 4 (*n* = 20) and Week 8 (*n* = 20), respectively. At Week 12 (*n* = 4), the reduction in the TWSTRS disability score (mean ± SD) in Group A was −3.8 ± 5.1.

#### 3.6.5. Reduction in the TWSTRS Pain Score (Group A)

In Group A, the reduction (change from the first injection baseline) in the TWSTRS pain score (mean ± SD) observed at Week 4 (*n* = 7) and Week 8 (*n* = 6) for botulinum toxin-naïve patients was −1.36 ± 2.9 and −2.54 ± 3.6, respectively, during the first injection cycle (Figure 4c), while in patients with previous botulinum toxin treatment, it was −2.59 ± 4.6 and −1.68 ± 4.1 at Week 4 (*n* = 20) and Week 8 (*n* = 20), respectively. At Week 12 (*n* = 4), the reduction in the TWSTRS pain score (mean ± SD) was −3.06 ± 2.5.

#### 3.6.6. CD Impact Profile (Group A)

In Group A, the mean change from the first injection baseline in the CDIP-58 score (mean ± SD) observed at Week 4 (*n* = 26) and Week 8 (*n* = 26) was −7.07 ± 11.5 and −3.03 ± 14.7, respectively, during the first injection cycle (Table S4).

#### 3.6.7. PEGR (Group A)

In Group A, the PEGR score (mean ± SD) at the first injection was 0.7 ± 1.29 (*n* = 27), and this value remained stable or showed a slightly upward trend across successive injection cycles, reaching 1.3 ± 1.39 (*n* = 15) by the sixth injection cycle. This indicated an improvement in CD symptoms as assessed by the patients (Table S5). The PEGR score (mean ± SD) during the first injection cycle was 1.1 ± 0.4 for botulinum toxin-naïve patients (*n* = 7) and 0.6 ± 1.5 for patients with previous botulinum toxin treatment.

#### 3.6.8. Subscale D of the Modified Tsui Scale (Head Tremor Assessment) (Group A)

At baseline, subscale D of the Modified Tsui Scale (mean ± SD) in Group A accompanied by head tremor was 1.5 ± 1.0 (*n* = 16) (botulinum toxin-naïve: 1.0 ± 0.0 [*n* = 2]; previous botulinum toxin treatment: 1.6 ± 1.1 [*n* = 14]). After administration of incobotulinumtoxinA, the reduction in subscale D of the Modified Tsui Scale (mean ± SD) in Group A at Week 4 (*n* = 16) and Week 8 (*n* = 16) was −0.4 ± 0.6 and −0.4 ± 1.0, respectively, during the first injection cycle. At Week 4, the reduction in the subscale D of the Modified Tsui Scale (mean ± SD) at the second, third, fourth, fifth, sixth, and seventh injections was −0.7 ± 0.9, −0.8 ± 0.9, −0.7 ± 0.9, −0.7 ± 0.9, −0.9 ± 1.0, and −0.7 ± 0.6, respectively (Table S6).

### 3.7. Exploratory Assessment of Patients with Tardive Dyskinesia (Cervical) and Tardive CD (Group B)

Baseline characteristics in Group B are presented in Table 1. Formal efficacy analysis was not performed for Group B because the sample size (*n* = 3) was insufficient to draw meaningful clinical conclusions. Descriptive observations indicated safety findings consistent with Group A, which are detailed in Section 3.9.

### 3.8. Subgroup Analysis (Group A)

**Figure 5:**
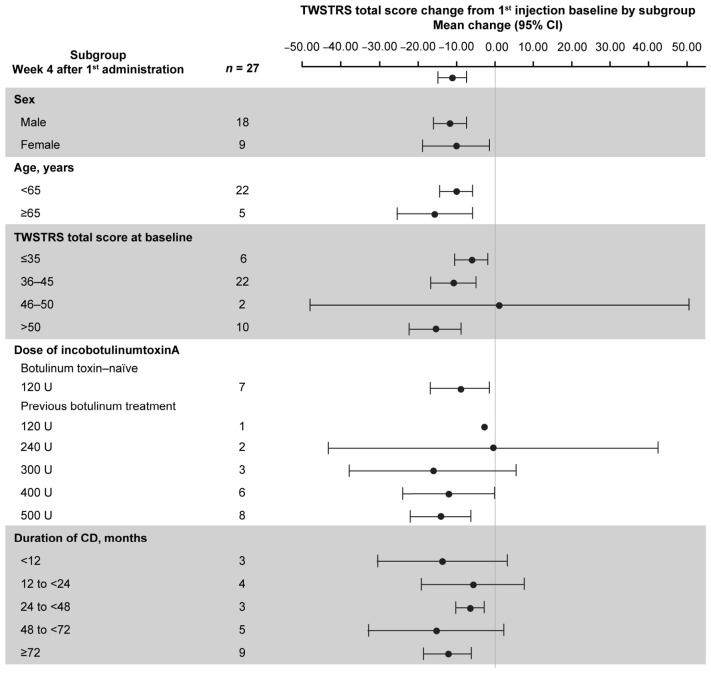
Subgroup analysis of patients with CD in Group A. CD, cervical dystonia; CI, confidence interval; TWSTRS, Toronto Western Spasmodic Torticollis Rating Scale.

Regardless of sex, age, TWSTRS total score at baseline, initial dose, and duration of CD, the first injection of incobotulinumtoxinA showed a consistent trend toward symptom amelioration in patients with CD in Group A (Figure 5). In botulinum toxin-naïve patients (*n* = 7), administration of 120 U demonstrated efficacy. In 20 patients with previous botulinum treatment, doses of 120 U (*n* = 1) and 240 U (*n* = 2) were less prevalent. While the very small sample sizes in certain subgroups limit formal statistical interpretation, these descriptive findings suggest that the therapeutic effect is maintained across the diverse clinical profiles within Group A.

### 3.9. Safety

In the primary evaluation group (Group A), AEs were reported in 21 (77.8%) patients, and related AEs were observed in 13 (48.1%) patients (Table 3). The most common related AEs in Group A were dysphagia (9 [33.3%]) and muscular weakness (6 [22.2%]) (Table S7). In the exploratory cohort (Group B), all 3 patients (100.0%) experienced AEs, with 1 patient (33.3%) reporting a related AE (dysphagia). Descriptive safety findings in this cohort also included influenza, nasopharyngitis, and dental caries (Table S8).

All cases of dysphagia recovered during the study period and were transient. Across the entire study population (*N* = 30), there was no observed trend of increased incidence of AEs or side effects with higher doses, and no notable trends were seen in AE incidence per preferred term at the upper dose limit of 500 U (Table S9). Serious AEs were reported in 1 (3.3%) patient overall, but no AE led to death, discontinuation, or serious related AE (Table S10). Furthermore, across the study population, AE incidence following the second injection varied by interval: 55.6% (10/18) for ≥6 to <8 weeks, 59.3% (16/27) for ≥8 to <12 weeks, and 60.0% (6/10) for ≥12 weeks. No apparent trend toward higher AE incidence was noted in patients with shorter intervals (<8 weeks).

## 4. Discussion

This study represents the first evaluation of the efficacy and safety of incobotulinumtoxinA for the treatment of CD in Japan, utilizing a tailored dosing strategy that incorporates high doses up to 500 U and flexible injection intervals as short as 6 weeks. As part of the clinical development program, this study was conducted following regulatory agreement in Japan. Given the rarity of CD in Japan (estimated at approximately 2000 patients) [38] and the ethical challenges of using a placebo or active comparator when effective treatments are available, a single-arm design was adopted. While this open-label, single-arm design represents a recognized limitation that prevents direct comparison with placebo effects or natural symptom fluctuations, it was considered an appropriate approach in this clinical context. The focus on Japanese patients is particularly important, as most previous studies have been conducted in Western populations. The results demonstrated that this tailored approach markedly improved motor symptoms, functional disability, pain, and QoL in Japanese patients. The primary endpoint—change in the TWSTRS total score from baseline to Week 4—met the prespecified efficacy threshold. Graphical plain language summaries of the study, presented in English language and Japanese language, are presented as Figure S1 and Figure S2, respectively.

Botulinum toxin injections are widely recognized as the first-line treatment for CD, and several formulations, including onabotulinumtoxinA and abobotulinumtoxinA, are commonly used. Previous randomized controlled trials (ClinicalTrials.gov identification number: NCT00407030) [16] have shown that incobotulinumtoxinA up to 240 U improves TWSTRS scores and has comparable efficacy and safety to other botulinum toxin formulations [15]. Across these treatments, dysphagia and muscle weakness have been consistently reported as the most common treatment-related AEs [16,17,19]. In contrast with previous studies conducted at doses up to 240 U, our study explored a tailored dosing strategy with doses up to 500 U, providing descriptive data on the potential clinical utility of higher-dose administration in Japanese clinical practice.

A key strength of this study is the utilization of multiple outcome measures, including both clinician-rated and patient-reported outcomes (TWSTRS subscales, CDIP-58, and PEGR), which provided a comprehensive evaluation of treatment effectiveness. Consistent improvement was observed across multiple injection cycles. This study also provides evidence for the efficacy of botulinum toxin therapy in head tremor, an area with limited evidence, with reductions observed in subscale D of the Modified Tsui Scale. Importantly, no refractory cases were identified, including no secondary treatment failures suggestive of neutralizing antibodies, consistent with the low immunogenicity of incobotulinumtoxinA [39].

Compared with previous studies [16], patients in this study had longer disease duration and more severe baseline symptoms, and the inclusion of both naïve and previously treated patients reflects real-world clinical practice. In this context, incobotulinumtoxinA was associated with improvement in Group A patients, with results broadly comparable to previous studies despite differences in design and dosing strategies, providing supportive evidence for the clinical utility of this tailored strategy in a more refractory population. Further details of the treatment effect are provided by the change in the TWSTRS total score at Week 4 (LSM −11.0; 95% CI −14.6 to −7.3), meeting the prespecified efficacy criteria. The magnitude of improvement was broadly comparable with that reported in previous studies [16], although direct comparisons are limited by differences in study design.

Results of TWSTRS scores at 48 weeks of repeated administration suggest a potential cumulative effect with continued treatment. Improvements in CDIP-58 and PEGR further support the clinical relevance of these findings, including benefits in nonmotor symptoms and QoL [40].

Evidence for improvement in head tremor was also observed; however, given the limited available data and the uncontrolled study design, these findings should be interpreted as exploratory.

Subgroup analysis suggested consistent trends across patient groups; however, some subgroups were small, and findings should be interpreted as exploratory. The heterogeneous population and flexible dosing (120–500 U) and intervals likely contributed to variability but also reflect real-world practice. In this context, the study population included a wide range of clinical presentations, including both botulinum toxin-naïve and previously treated patients. Some dose subgroups, particularly those receiving 120 U or 240 U, included relatively few patients, limiting the ability to evaluate their efficacy within this study. However, the efficacy of these doses has been well established in previous placebo-controlled trials [16], and similar trends were observed in botulinum toxin-naïve patients in this study. In addition, prior comparative studies have demonstrated that incobotulinumtoxinA has efficacy similar to other botulinum toxin formulations [15].

Furthermore, the subgroup analysis suggested that efficacy was not clearly dependent on the total dose administered, although these findings should be interpreted with caution given the exploratory nature and small sample sizes of some subgroups. Based on the dose administered by target muscle (Table S2), relatively larger muscles received higher doses. Higher dose administration allows more comprehensive targeting of multiple causative muscles, supporting the clinical practice of tailored dosing strategy based on muscle size and symptom severity, with no observed trend of increased safety risk in this study.

In Group A, the most frequently observed AEs were dysphagia and muscular weakness, consistent with previous findings [16,17,19]. In the exploratory cohort (Group B), influenza, nasopharyngitis, dental caries, and dysphagia were each reported as AEs in 33.3% of patients, whereas dysphagia (33.3%) was reported as a related AE. Group B in this study consisted of patients with tardive dyskinesia (cervical) or tardive dystonia (cervical), a population excluded from previous international trials, making direct comparisons difficult.

Overall, the incidence of AEs during the first injection cycle was 33.3%, with related AEs at 26.7%, which was numerically lower than that reported in international studies [16,17,19]. Nevertheless, the overall safety profile in this study showed no clear differences compared with international studies [16,17,19].

Dysphagia warrants particular consideration given its relatively high incidence. Importantly, all cases of dysphagia observed in this study were mild-to-moderate in severity, transient in nature, and resolved completely without sequelae. No patients discontinued the study due to dysphagia, and no serious related AEs, severe AEs, or deaths were reported.

The observed incidence of dysphagia may be influenced by multiple factors, including higher total doses, injections into deep cervical paraspinal muscles, and the inclusion of refractory patients with longer disease duration and greater disease severity. Although higher-dose administration and shorter injection intervals were permitted in this study, no clear trend toward an increased incidence of AEs was observed in patients receiving injections at intervals shorter than 8 weeks. Similarly, increased doses, including doses up to 500 U, were not clearly associated with a higher overall incidence of AEs or with specific AE patterns by preferred term.

Taken together, these findings indicate that while dysphagia represents an important safety consideration in the context of high-dose treatment, the events observed in this study were considered clinically manageable. Careful muscle selection, individualized dose optimization, and close clinical monitoring remain essential when employing higher-dose or more frequent dosing strategies.

A known challenge in botulinum toxin therapy is secondary nonresponse, which has been associated with the development of neutralizing antibodies [41]. In this study, no discontinuation due to nonresponse was observed, consistent with previous evidence indicating low immunogenicity of incobotulinumtoxinA [39]. The study design allowed for a tailored dosing strategy, enabling the inclusion of patients with more complex clinical characteristics. While patients with prior treatment often required higher doses, botulinum toxin-naïve patients responded favorably to lower initial doses, supporting individualized dose optimization based on treatment history and disease severity.

This study has some limitations that must be acknowledged. First, the single-arm design limits the ability to draw definitive conclusions about the efficacy of incobotulinumtoxinA compared with placebo or other active treatments and prevents differentiation of the treatment effect from placebo response, regression to the mean, or natural symptom fluctuations. Second, the relatively small sample size may limit the generalizability of the findings to a broader population of patients with CD. Furthermore, the very small sample size of Group B (*n* = 3) represents a major limitation, making findings for tardive symptoms purely descriptive and not generalizable. Third, the observation period was limited to 48 weeks, highlighting the need for further research to assess the long-term safety and effectiveness of incobotulinumtoxinA in real-world clinical settings. Fourth, the study used a variable-dose regimen, and neither stratification nor formal dose–response analysis based on changes from each pre-continuation assessment was conducted; therefore, the therapeutic effect at each dose level could not be accurately determined. Fifth, missing data represents a limitation of this study. Although observed case analysis and the baseline-observation-carried-forward approach were used, these methods may introduce potential bias. However, the primary objective was descriptive confirmation of safety and efficacy across dosing conditions rather than a formal hypothesis testing between dose levels. Importantly, consistent efficacy and safety trends were observed across multiple clinical outcomes and repeated injection cycles, which lends support to the overall interpretation of the findings despite these limitations.

To better isolate and quantify the dose–response and safety relationship of higher-dose incobotulinumtoxinA, future clinical trials should incorporate standardized dose tiers within a controlled framework. For example, a randomized parallel-group design with prespecified fixed total-dose arms (e.g., low-, mid-, and high-dose tiers) could enable direct comparisons across dose levels, whereas a mandatory intra-patient titration design with predefined escalation steps and response- and safety-based criteria could characterize within-patient dose–response more precisely. Such approaches would help distinguish the independent therapeutic and safety profiles of the upper dose tier (e.g., 500 U) from those of lower-dose tiers.

## 5. Conclusions

The results of this prospective, multicenter, open-label, single-arm study conducted in Japan were generally consistent with findings from international studies. This study suggests that a tailored dosing strategy of incobotulinumtoxinA, incorporating higher doses (up to 500 U) and flexible injection intervals (as short as 6 weeks), was associated with improvements in CD symptoms without the emergence of new safety concerns. Across a range of doses and injection intervals tailored to patient needs, incobotulinumtoxinA was associated with improvements in motor symptoms, ADL, and QoL. Nevertheless, given the single-arm design and other study limitations, these findings should be interpreted with caution. Overall, the results suggest the potential role of incobotulinumtoxinA as a well-tolerated treatment option for CD in Japanese clinical practice.

## Figures and Tables

**Table 1:** Baseline demographics and characteristics of patients with idiopathic CD (Group A) and exploratory tardive cases (Group B [exploratory cohort]) (full analysis set).

Characteristic	Group A (*N* = 27)	Group B (Exploratory Cohort) (*N* = 3)	Total (*N* = 30) ^a^
Japanese, *n* (%)	27 (100.0)	3 (100.0)	30 (100.0)
Sex, *n* (%)			
Male	18 (66.7)	2 (66.7)	20 (66.7)
Female	9 (33.3)	1 (33.3)	10 (33.3)
Age, years			
Mean ± SD	54.5 ± 11.27	48.3 ± 13.65	53.9 ± 11.41
<65, *n* (%)	22 (81.5)	3 (100.0)	25 (83.3)
≥65, *n* (%)	5 (18.5)	0 (0.0)	5 (16.7)
Type of CD ^b^, *n* (%) ^c^			
Rotation	27 (100.0)	3 (100.0)	30 (100.0)
Retrocollis	11 (40.7)	1 (33.3)	12 (40.0)
Anterocollis	13 (48.1)	1 (33.3)	14 (46.7)
Laterocollis	25 (92.6)	3 (100.0)	28 (93.3)
Shift	19 (70.4)	2 (66.7)	21 (70.0)
Shoulder elevation	23 (85.2)	3 (100.0)	26 (86.7)
Scoliosis	8 (29.6)	1 (33.3)	9 (30.0)
Head tremor	16 (59.3)	1 (33.3)	17 (56.7)
Duration of CD ^d^ (months)			
Mean ± SD	92.58 ± 123.9	111.17 ± 31.0	94.65 ± 117.0
Median	54.62	124.69	60.36
Treatment history for CD with botulinum toxin preparation, *n* (%)			
Botulinum toxin-naïve	7 (25.9)	0 (0.0)	7 (23.3)
Previous botulinum toxin treatment	20 (74.1)	3 (100.0)	23 (76.7)
Dose of onabotulinumtoxinA at the last dose ^e^, *n* (%)			
<120 U	4 (20.0)	1 (33.3)	5 (21.7)
≥120 U to <240 U	8 (40.0)	0 (0.0)	8 (34.8)
≥240 U	7 (35.0)	2 (66.7)	9 (39.1)
Dose of rimabotulinumtoxinB at the last dose ^e^, *n* (%)			
<5000 U	1 (5.0)	0 (0.0)	1 (4.3)
≥5000 U to <10,000 U	0 (0.0)	0 (0.0)	0 (0.0)
≥10,000 U	0 (0.0)	0 (0.0)	0 (0.0)
Presence of complications, *n* (%)			
No	9 (33.3)	0 (0.0)	9 (30.0)
Yes	18 (66.7)	3 (100.0)	21 (70.0)
TWSTRS total score, mean ± SD			
All patients	46.24 ± 10.33	45.75 ± 1.32	46.19 ± 9.78
Botulinum toxin-naïve	47.07 ± 7.45	NA	NA
Previous botulinum toxin treatment	45.95 ± 11.32	45.75 ± 1.32	NA
TWSTRS severity score, mean ± SD			
All patients	23.0 ± 3.19	24.7 ± 2.08	NA
Botulinum toxin-naïve	22.3 ± 3.30	NA	NA
Previous botulinum toxin treatment	23.2 ± 3.21	24.7 ± 2.08	NA
Severity of CD, *n* (%)			
Mild: TWSTRS severity score is from 0 to 15	0 (0.0)	0 (0.0)	0 (0.0)
Moderate: TWSTRS severity score is from 16 to 21	8 (29.6)	0 (0.0)	8 (26.7)
Severe: TWSTRS severity score is from 22 to 35	19 (70.4)	3 (100.0)	22 (73.3)
TWSTRS disability score, mean ± SD			
All patients	12.8 ± 4.72	12.7 ± 2.08	NA
Botulinum toxin-naïve	14.1 ± 3.48	NA	NA
Previous botulinum toxin treatment	12.4 ± 5.08	12.7 ± 2.08	NA
TWSTRS pain score, mean ± SD			
All patients	10.46 ± 4.29	8.42 ± 3.01	NA
Botulinum toxin-naïve	10.64 ± 2.66	NA	NA
Previous botulinum toxin treatment	10.40 ± 4.79	8.42 ± 3.01	NA
CDIP-58, mean ± SD			
All patients	41.51 ± 20.42	54.17 ± 13.32	NA
Subscale D of the Modified Tsui Scale, mean ± SD			
All patients	1.5 ± 1.0	4.0	1.6 ± 1.2
Botulinum toxin-naïve	1.0 ± 0.0	NA	NA
Previous botulinum toxin treatment	1.6 ± 1.1	4.0	NA

^a^ The total population for TWSTRS severity, TWSTRS disability, TWSTRS pain scores, or CDIP-58 scores was not calculated. ^b^ The type of CD was assessed and determined by the investigator based on the patients’ CD symptoms. ^c^ Multiple entries possible. ^d^ Duration of CD (months) = [date of the 1st injection—date of clinical diagnosis for CD]/30.5 ^e^ Denominator is the number of patients for whom treatment history for CD with botulinum toxin preparation is “Yes.” CD, cervical dystonia; CDIP-58, Cervical Dystonia Impact Profile-58; NA, not applicable; SD, standard deviation; TWSTRS, Toronto Western Spasmodic Torticollis Rating Scale.

**Table 2:** Dosage of incobotulinumtoxinA (full analysis set).

	1st Injection Cycle	2nd Injection Cycle	3rd Injection Cycle	4th Injection Cycle	5th Injection Cycle	6th Injection Cycle	7th Injection Cycle
Total Dose volume (U) ^a^							
*n*	30	30	29	27	25	17	3
Mean ± SD	325.3 ± 156.4	362.3 ± 141.2	372.4 ± 141.4	378.0 ± 138.6	412.4 ± 121.4	428.2 ± 111.1	500.0 ± 0.0
Min	120	140	100	95	180	200	500
Median	400.0	375.0	400.0	400.0	500.0	500.0	500.0
Max	500	500	500	500	500	500	500
Botulinum toxin-naïve							
*n*	7	7	6	6	5	5	0
Mean ± SD	120.0 ± 0.0	202.1 ± 43.8	221.7 ± 98.9	225.8 ± 100.6	292.0 ± 122.1	316.0 ± 128.4	-
Min	120	140	100	95	200	200	-
Median	120.0	220.0	200.0	220.0	240.0	240.0	-
Max	120	240	400	400	500	500	-
Previous botulinum toxin treatment							
*n*	23	23	23	21	20	12	3
Mean ± SD	387.8 ± 121.5	411.1 ± 123.0	411.7 ± 124.1	421.4 ± 116.2	442.5 ± 103.4	475.0 ± 62.2	500.0 ± 0.0
Min	120	150	150	150	180	300	500
Median	400.0	500.0	500.0	500.0	500.0	500.0	500.0
Max	500	500	500	500	500	500	500
Group A							
*n*	27	27	26	24	22	15	3
Botulinum toxin-naïve (first injection cycle) ^b^							
120 U	7 (25.9)	-	-	-	-	-	-
Previous botulinum toxin treatment (first injection cycle) ^b^							
120 U	1 (3.7)	-	-	-	-	-	-
240 U	2 (7.4)	-	-	-	-	-	-
300 U	3 (11.1)	-	-	-	-	-	-
400 U	6 (22.2)	-	-	-	-	-	-
500 U	8 (29.6)	-	-	-	-	-	-
Group B (exploratory cohort)							
*n*	3	3	3	3	3	2	0
Previous botulinum toxin treatment (first injection cycle) ^b^							
120 U	1 (33.3)	-	-	-	-	-	-
240 U	0 (0.0)	-	-	-	-	-	-
300 U	0 (0.0)	-	-	-	-	-	-
400 U	1 (33.3)	-	-	-	-	-	-
500 U	1 (33.3)	-	-	-	-	-	-

Results are presented as *n* (%) unless otherwise specified. Results presented in the table are Dose volume, unit U. ^a^ Dose volume (U) for each injection cycle = dose volume in each injection cycle. ^b^ 1st injection cycle indicates the main cycle. Max, maximum; Min, minimum; SD, standard deviation.

**Table 3:** Number (%) of patients with AEs during treatment with incobotulinumtoxinA for a 48-week study cycle (safety analysis set).

	Group A (*n* = 27)	Group B (Exploratory Cohort) (*n* = 3)	Total (*N* = 30)
Any AE	21 (77.8)	3 (100.0)	24 (80.0)
Any related AE	13 (48.1)	1 (33.3)	14 (46.7)
Leading to death			
AE	0 (0.0)	0 (0.0)	0 (0.0)
Related AE	0 (0.0)	0 (0.0)	0 (0.0)
Serious			
AE	1 (3.7)	0 (0.0)	1 (3.3)
Related AE	0 (0.0)	0 (0.0)	0 (0.0)
Leading to discontinuation of the study drug			
AE	0 (0.0)	0 (0.0)	0 (0.0)
Related AE	0 (0.0)	0 (0.0)	0 (0.0)
Maximum severity			
AE ^a^			
Mild	12 (44.4)	3 (100.0)	15 (50.0)
Moderate	9 (33.3)	0 (0.0)	9 (30.0)
Severe	0 (0.0)	0 (0.0)	0 (0.0)
Related AE ^a^			
Mild	7 (25.9)	1 (33.3)	8 (26.7)
Moderate	6 (22.2)	0 (0.0)	6 (20.0)
Severe	0 (0.0)	0 (0.0)	0 (0.0)

AEs are defined as any AE, regardless of relationship to the study drug. Percentages are based on the total number of patients in the safety set for each group. ^a^ Patients with one or more AEs within a level of MedDRA term were counted only once in that level using the most severe incident. AE, adverse event; MedDRA, Medical Dictionary for Regulatory Activities.

## Data Availability

Individual participant data underlying the results reported in this study are not publicly available. These data are owned by Teijin Pharma and are subject to legal and contractual restrictions.

## Supplementary Materials

The following supporting information can be downloaded at: https://www.mdpi.com/article/10.3390/neurolint18070136/s1, Figure S1: Graphical plain language summary (English), Figure S2: Graphical plain language summary (Japanese); Table S1: Dosing interval of incobotulinumtoxinA (full analysis set); Table S2: Dose of incobotulinumtoxinA by target muscle group at different time points; Table S3: TWSTRS Total score change from the 1st injection cycle baseline by visit and muscle group (Group A); Table S4: CDIP-58 change from the 1st injection cycle baseline by visit (full analysis set); Table S5: PEGR score trends across injection cycles by treatment group (full analysis set); Table S6: Change in subscale D of the Modified Tsui Scale from the 1st injection baseline in patients with onset of head tremor at each injection cycle by visit (full analysis set); Table S7: Related AEs by SOC and PT (safety analysis set); Table S8: AEs that occurred in ≥2 patients in the pooled population (safety analysis set); Table S9: AEs by dose of incobotulinumtoxinA; Table S10: AEs during treatment with incobotulinumtoxinA at each stage of administration (safety analysis set). Table S11: The details of the institutional review board approvals for all 15 participating institutions (Protocol number: NT 201C-301).

## Abbreviations

The following abbreviations are used in this manuscript:

ADL	Activities of daily living
AE	Adverse event
CD	Cervical dystonia
CDIP-58	Cervical Dystonia Impact Profile-58
CI	Confidence interval
FAS	Full analysis set
LSM	Least squares mean
MedDRA	Medical Dictionary for Regulatory Activities
PEGR	Patient Evaluation of Global Response
QoL	Quality of life
SD	Standard deviation
SNAP-25	Synaptosomal-associated protein, 25 kDa
SE	Standard error
TWSTRS	Toronto Western Spasmodic Torticollis Rating Scale
US	United States
