# Supplementary material for: Efficacy and Safety of a Tailored Dosing Strategy with High-Dose IncobotulinumtoxinA at Flexible Injection Intervals for Cervical Dystonia: An Open-Label, Uncontrolled, Single-Arm Study in Japan
Source: Neurol Int. 2026 Jul 15;18(7):136. doi: 10.3390/neurolint18070136 (PMC13414479; doi:10.3390/neurolint18070136)
Supplement: Supplementary file 1 [file neurolint-18-00136-s001.zip › Figure S1_English.pdf]

Efficacy and Safety of a Tailored Dosing Strategy with High-Dose IncobotulinumtoxinA at Flexible Injection Intervals for Cervical Dystonia: An Open-Label, Uncontrolled, Single-Arm Study in Japan

What is cervical dystonia?

Cervical dystonia, also known as spasmodic torticollis, is a neurological condition that causes the muscles in your neck to contract without your control. This leads to involuntary movements or abnormal postures of the head and neck, such as twisting, tilting, or jerking.

What was the main purpose of the study?

The study aimed to evaluate the efficacy and safety of a tailored dosing strategy using incobotulinumtoxinA, incorporating higher doses (up to 500 U) and flexible injection intervals (as short as 6 weeks), in Japanese patients with cervical dystonia.

How was this study performed?

This study was conducted at 15 hospitals and clinics across Japan. In total, 30 patients participated in the study.

30 Japanese patients

Group A

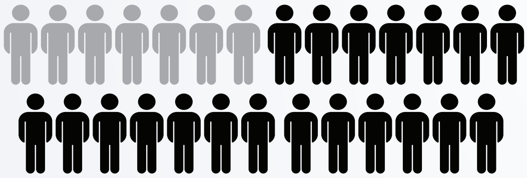

27 patients with cervical dystonia

- Average age: 54.5 years
- Average TWSTRS total score: 46.24
- Average TWSTRS severity score: 23.0
- Average TWSTRS disability score: 12.8
- Average TWSTRS pain score: 10.46

Group B
(exploratory cohort)

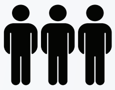

3 patients with tardive dyskinesia (cervical) or tardive dystonia (cervical)

- Average age: 48.3 years
- Average TWSTRS total score: 45.75
- Average TWSTRS severity score: 24.7
- Average TWSTRS disability score: 12.7
- Average TWSTRS pain score: 8.42

Botulinum toxin–naïve
Previous botulinum toxin treatment

What is tardive dyskinesia (cervical), and what is tardive dystonia (cervical)?

People may experience symptoms of spasmodic torticollis because of the drugs they take. Such conditions are called tardive dyskinesia (cervical) or tardive dystonia (cervical).

IncobotulinumtoxinA injections

<Dose>

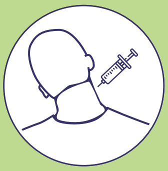

Botulinum toxin–naïve:

- **1st injection:** 120 U
- **2nd injection:** flexible doses (up to 240 U)
- **3rd and later injections:** flexible doses (up to 500 U)

Previous botulinum toxin treatment:

- **1st injection:** 120, 240, 300, 400, or 500 U
- **2nd injection:** flexible doses (up to 500 U)
- **3rd and later injections:** flexible doses (up to 500 U)

<Interval>

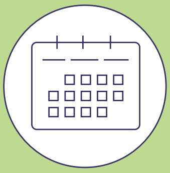

- **2nd injection:** at least 8-week interval after the 1st injection
- **3rd and later injections:** at least 6-week intervals

The dose and dosing interval were determined by the trial doctor based on symptoms and previous botulinum toxin treatment.

Patients were followed up for 48 weeks, and the trial doctors used standard clinical scales (TWSTRS and subscale D of the Modified Tsui Scale) and patient questionnaires, such as the Cervical Dystonia Impact Profile-58 (CDIP-58), to assess changes in neck muscle spasms and overall well-being. Safety was monitored throughout the study.

What is the TWSTRS score?

TWSTRS is a clinical scale to measure how much cervical dystonia symptoms affect an individual. It includes three subscales of severity (physical symptoms), disability (impact on work and daily activities), and pain in the head and neck. Each subscale is scored individually: severity (0–35), disability (0–30), and pain (0–20). The total score is calculated by adding the scores of the three subscales (0–85). Higher scores mean more severe cervical dystonia symptoms.

What is the CDIP-58 score?

CDIP-58 is a self-administered, 58-item questionnaire consisting of 8 areas: head and neck symptoms, pain and discomfort, upper limb activities, gait, sleep, irritability, mood, and psychosocial functioning. Patients assessed their symptoms on a 5-point scale. Higher scores indicate greater difficulty or distress caused by the symptoms.

What is subscale D of the Modified Tsui Scale?

Subscale D of the Modified Tsui Scale was used to assess the severity and duration of the head tremor. Each parameter was scored separately, and the final score was calculated by adding the two values together. Higher scores indicate more severe and persistent head tremor.

What were the main results of the study?

What were the main efficacy results?

Patients showed an improvement in neck muscle spasms (TWSTRS total score) 4 weeks after the 1st injection. The observed change was considered clinically meaningful.

Repeated injections of incobotulinumtoxinA were associated with sustained improvements in cervical dystonia. In addition, patients reported improvements in self-assessed symptoms with repeated incobotulinumtoxinA injections. The treatment was also associated with reductions in head tremor, a key symptom affecting daily functions in patients with cervical dystonia.

Doctor's assessment

- Improvement in TWSTRS score (severity, disability, pain, and total)
- Improvement in subscale D of the Modified Tsui Scale

Patient's assessment

- Improvement in CDIP-58 score

What were the adverse events and treatment-related adverse events reported during the study?

All adverse events reported were mild to moderate. During the study, the following adverse events were observed. There were no serious treatment-related adverse events during the study. None of the patients stopped incobotulinumtoxinA treatment because of adverse events.

Group A

Adverse events (in 2 or more patients)

- Difficulty swallowing (dysphagia): **9 patients (33.3%)**
- Muscular weakness: **6 patients (22.2%)**
- Common cold (nasopharyngitis): **6 patients (22.2%)**
- COVID-19: **3 patients (11.1%)**
- Bruise (contusion): **2 patients (7.4%)**

Treatment-related adverse events (in 2 or more patients)

- Difficulty swallowing (dysphagia): **9 patients (33.3%)**
- Muscular weakness: **6 patients (22.2%)**

Group B (exploratory cohort)

Adverse events

- Flu (influenza): **1 patient (33.3%)**
- Tooth decay (dental caries): **1 patient (33.3%)**
- Common cold (nasopharyngitis): **1 patient (33.3%)**
- Difficulty swallowing (dysphagia): **1 patient (33.3%)**

Treatment-related adverse event

- Difficulty swallowing (dysphagia): **1 patient (33.3%)**

What is an adverse event, and what is a treatment-related adverse event?

An adverse event is any medical problem that a patient has during a trial. A treatment-related adverse event is an adverse event reported by the trial doctor as possibly related to trial treatment. An adverse event or treatment-related adverse event is considered serious when it is life-threatening, causes lasting problems, or requires hospital care.

How has this study helped patients and trial doctors?

This study suggests that a tailored dosing strategy of incobotulinumtoxinA, incorporating higher doses (up to 500 U) and flexible injection intervals (as short as 6 weeks), was associated with improvements in cervical dystonia symptoms and was generally well tolerated in Japanese patients.

Where can I learn more about this study?

You can find more information about this trial on the following website: https://jrct.mhlw.go.jp/en-latest-detail/jRCT2031230690

Full study title: An open-label, uncontrolled, single-arm study of NT 201 in patients with cervical dystonia

Clinical trial ID: jRCT2031230690

Trial sponsor: Teijin Pharma Limited
