# Supplementary material for: Efficacy and Safety of a Tailored Dosing Strategy with High-Dose IncobotulinumtoxinA at Flexible Injection Intervals for Cervical Dystonia: An Open-Label, Uncontrolled, Single-Arm Study in Japan
Source: Neurol Int. 2026 Jul 15;18(7):136. doi: 10.3390/neurolint18070136 (PMC13414479; doi:10.3390/neurolint18070136)
Supplement: Supplementary file 1 [file neurolint-18-00136-s001.zip › Figure S2_Japanese.pdf]

痙性斜頸の治療における高用量インコボツリヌストキシンAを用いた柔軟な投与間隔による個別化投与戦略の有効性および安全性：国内非盲検・非対照・単群試験

痙性斜頸とはどんな病気ですか？

痙性斜頸（けいせいしゃけい）は、頸部（けいぶ）ジストニアとも呼ばれ、首や肩の周囲の筋肉が自分の意思とは関係なく緊張、収縮して不自然な姿勢になってしまう病気です。
痙性斜頸は、頭が横を向く、頭が横に倒れる、頭がふるえるなどの症状を引き起こします。

この治験の目的は何ですか？

日本人の痙性斜頸の患者さんに対して、インコボツリヌストキシンAという治験薬を使用しました。
患者さん一人ひとりの症状に合わせて、注射量（最大500単位）や注射間隔（最短6週間）を調整しながら注射した場合の治験薬の効果、治験薬の安全性を評価しました。

この治験はどのように行われましたか？

この治験は、日本全国の15の病院およびクリニックで行われました。30名の患者さんが参加しました。

日本人の患者さん30名

グループA

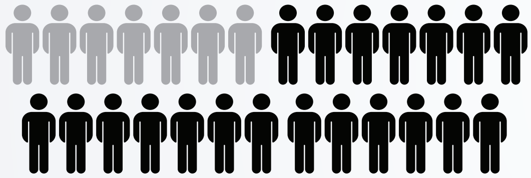

痙性斜頸の患者さん27名

- 平均年齢：54.5歳
- 平均のTWSTRS合計スコア：46.24
- 平均のTWSTRS重症度スコア：23.0
- 平均のTWSTRS機能障害スコア：12.8
- 平均のTWSTRS疼痛スコア：10.46

グループB（少人数の参考グループ）

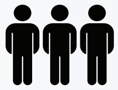

遅発性ジスキネジア（頸部）または
遅発性ジストニア（頸部）の患者さん3名

- 平均年齢：48.3歳
- 平均のTWSTRS合計スコア：45.75
- 平均のTWSTRS重症度スコア：24.7
- 平均のTWSTRS機能障害スコア：12.7
- 平均のTWSTRS疼痛スコア：8.42

ボツリヌス治療を受けたことがない　　ボツリヌス治療を受けたことがある

遅発性（ちはつせい）ジスキネジア（頸部）、遅発性ジストニア（頸部）とはどんな病気ですか？

服薬しているお薬が原因で、痙性斜頸の症状が現れることがあります。
このような状態を、遅発性ジスキネジア（頸部）または遅発性ジストニア（頸部）と言います。

インコボツリヌストキシンAの注射方法

<注射量>

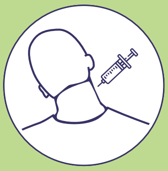

ボツリヌス治療を受けたことがない患者さん:
- 1回目:120単位
- 2回目:240単位を超えないように設定
- 3回目以降:500単位を超えないように設定

ボツリヌス治療を受けたことがある患者さん:
- 1回目:120、240、300、400、500単位のいずれか
- 2回目:500単位を超えないように設定
- 3回目以降:500単位を超えないように設定

<注射間隔>

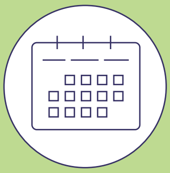

- 2回目:1回目から少なくとも8週間以上あけて注射
- 3回目以降:少なくとも6週間以上あけて注射

注射する量や注射間隔は、患者さんそれぞれの痙性斜頸の症状に応じて、医師が決めました。

患者さんは、48週間にわたって治験に参加しました。
医師は、TWSTRSスコアやModified Tsui scaleのsub scale D、質問票への患者さんの回答(CDIP-58)などを用いて、痙性斜頸の症状の変化を確認しました。
また、安全性を確認しました。

TWSTRSスコアとは何ですか?

患者さんの痙性斜頸の「症状の重さ(重症度)」を評価する方法です。
痙性斜頸によるからだの症状(重症度、0~35点)、仕事や生活への影響(機能障害、0~30点)、および頭や首の痛み(疼痛、0~20点)をそれぞれ評価します。
合計スコアは、これらのスコアを足した値(0~85点)です。
スコアが高いほど、痙性斜頸の症状が悪いことを示します。

CDIP-58スコアとは何ですか?

58項目の質問票を使って、頭部の症状、痛みや不快な症状、両腕の活動性、歩行、睡眠、イラつき、気分、心理社会的機能の程度を患者さんが回答して評価する方法です。
8つの項目のそれぞれについて、5段階で評価します。
スコアが高いほど、痙性斜頸の症状による不便さや不快感が大きいことを示します。

Modified Tsui scaleのsub scale Dとは何ですか?

患者さんの頭のふるえの「症状の重さ(重症度)」と「症状が続く時間」をそれぞれ評価し、これらの値からスコアを求めます。
スコアが高いほど、頭のふるえの症状が悪く、長く続くことを示します。

この治験の主な結果は、どのようなものでしたか?

有効性の主な結果は、どうでしたか?

インコボツリヌストキシンAの1回目の注射から4週間後の時点で、痙性斜頸の症状(TWSTRS合計スコア)が改善することが示されました。

インコボツリヌストキシンAを繰り返し注射することによって、痙性斜頸の症状の改善が続くことが示されました。
また、質問票を用いた患者さん本人の評価によっても、痙性斜頸の症状が改善することが示されました。
頭のふるえも改善することが示されました。

医師による評価

- TWSTRSスコア(重症度、機能障害、疼痛および合計)の改善
- Modified Tsui scaleのsub scale Dの改善

患者さんによる評価

- CDIP-58スコアの改善

治験中に報告された有害事象と副作用はどのようなものでしたか?

治験中にあった有害事象と副作用は以下の通りでした。

この治験中に、重篤な副作用の報告はありませんでした。

有害事象のためにこの治験を中止した患者さんはいませんでした。

グループA

2名以上の報告があった有害事象

飲みこみづらい（嚥下障害）：9名（33.3%）

筋力低下：6名（22.2%）

かぜ（上咽頭炎）：6名（22.2%）

新型コロナウイルス感染症（COVID-19）：3名（11.1%）

打ち身（挫傷）：2名（7.4%）

2名以上の報告があった副作用

飲みこみづらい（嚥下障害）：9名（33.3%）

筋力低下：6名（22.2%）

グループB（少人数の参考グループ）

有害事象

インフルエンザ：1名（33.3%）

むし歯（齲歯）：1名（33.3%）

かぜ（上咽頭炎）：1名（33.3%）

飲みこみづらい（嚥下障害）：1名（33.3%）

副作用

飲みこみづらい（嚥下障害）：1名（33.3%）

有害事象とは何ですか?副作用とは何ですか?

治験期間中に参加者にみられた健康上の問題を、有害事象と言います。
有害事象のうち、治験を行った医師が「治験で受けた治療と関係がある」として報告したものを、副作用と言います。
有害事象および副作用は、生命に影響を及ぼす場合、問題を長い期間 引き起こす場合や入院して治療することが必要な場合には、重篤な有害事象、重篤な副作用と言います。

患者さんや医師にとって、この治験はどのように役立ちましたか?

日本人の痙性斜頸の患者さんに対して、首や肩のまわりにインコボツリヌストキシンAを注射して治療することは症状を改善し、多くの患者さんが無理なく続けられることが示されました。特に、患者さん一人ひとりの症状に合わせて、注射量（最大500単位）や注射間隔（最短6週間）を調整しながら注射した場合の有効性と安全性が示されました。

この治験の詳細を知るにはどうすればよいですか?

この治験に関する詳しい情報はこちらからご確認いただけます。

https://jrct.mhlw.go.jp/latest-detail/jRCT2031230690

治験の標題：痙性斜頸患者を対象としたNT 201の非盲検、非対照、単群試験

登録番号：jRCT2031230690

治験依頼者：帝人ファーマ株式会社
