# Supplementary material for: Efficacy and Safety of a Tailored Dosing Strategy with High-Dose IncobotulinumtoxinA at Flexible Injection Intervals for Cervical Dystonia: An Open-Label, Uncontrolled, Single-Arm Study in Japan
Source: Neurol Int. 2026 Jul 15;18(7):136. doi: 10.3390/neurolint18070136 (PMC13414479; doi:10.3390/neurolint18070136)
Supplement: Supplementary file 1 [file neurolint-18-00136-s001.zip › Tables S1-S11.pdf]

Supplementary Material

Table S1. Dosing interval of incobotulinumtoxinA (full analysis set).

Dosing interval (weeks)	Observed value				
	n	Mean±SD	Min	Median	Max
Group A					
Total	117	8.53±2.3	6.0	8.00	18.0
Between 1st and 2nd injection	27	8.89±1.3	8.0	8.71	12.0
Between 2nd and 3rd injection	26	8.39±2.5	6.0	8.00	17.0
Between 3rd and 4th injection	24	9.24±3.0	6.0	8.00	18.0
Between 4th and 5th injection	22	8.21±2.4	6.0	8.00	16.0
Between 5th and 6th injection	15	7.93±1.3	6.0	8.00	11.3
Between 6th and 7th injection	3	6.24±0.4	6.0	6.00	6.7
Botulinum toxin–naïve					
Total	29	8.61±1.8	6.0	8.00	14.0
Between 1st and 2nd injection	7	9.39±1.8	8.0	8.71	12.0
Between 2nd and 3rd injection	6	8.62±1.8	6.7	8.00	12.0
Between 3rd and 4th injection	6	8.50±2.8	6.0	8.00	14.0
Between 4th and 5th injection	5	8.06±1.5	6.0	8.00	10.3
Between 5th and 6th injection	5	8.20±0.4	8.0	8.00	9.0
Between 6th and 7th injection	0	-	-	-	-
Previous botulinum toxin treatment					
Total	88	8.51±2.4	6.0	8.00	18.0
Between 1st and 2nd injection	20	8.72±1.1	8.0	8.43	12.0
Between 2nd and 3rd injection	20	8.32±2.7	6.0	7.57	17.0
Between 3rd and 4th injection	18	9.48±3.1	6.0	9.07	18.0
Between 4th and 5th injection	17	8.26±2.6	6.0	8.00	16.0
Between 5th and 6th injection	10	7.80±1.6	6.0	7.93	11.3
Between 6th and 7th injection	3	6.24±0.4	6.0	6.00	6.7
Group B (exploratory cohort)					
Total (previous botulinum toxin treatment)	14	8.71±2.4	6.0	8.00	16.0
Between 1st and 2nd injection	3	9.33±2.3	8.0	8.00	12.0
Between 2nd and 3rd injection	3	10.67±4.6	8.0	8.00	16.0
Between 3rd and 4th injection	3	8.00±0.0	8.0	8.00	8.0
Between 4th and 5th injection	3	7.33±1.2	6.0	8.00	8.0
Between 5th and 6th injection	2	8.00±0.0	8.0	8.00	8.0

Dosing interval between (n)th and (n+1)th injection (weeks) = [date of (n+1)th injection - (n)th injection]/7.

If the (n+1)th injection was not administered, the treatment interval was not derived.

Treatment interval (total) refers to all calculated treatment intervals per injection cycle (weeks).

Max, maximum; Min, minimum; SD, standard deviation.

Table S2. Dose of incobotulinumtoxinA by target muscle group at different time points.

Muscle group [a]	1st injection	2nd injection	3rd injection	4th injection	5th injection	6th injection	7th injection
Sternocleidomastoid							
n	22	22	20	18	17	10	1
Mean±SD	49.8±32.7	58.4±35.1	60.0±31.7	60.8±35.3	56.8±33.9	66.5±31.5	75.0±(-)
Min	5	10	10	10	10	20	75
Median	50.0	50.0	50.0	50.0	50.0	55.0	75.0
Max	150	150	150	150	150	120	75
Trapezius							
n	11	13	12	13	11	9	3
Mean±SD	63.2±33.9	62.7±33.5	65.8±33.2	76.2±62.3	83.6±43.7	97.8±39.9	166.7±125.8
Min	30	25	40	20	40	50	50
Median	50.0	50.0	50.0	50.0	80.0	100.0	150.0
Max	150	150	150	250	170	150	300
Trapezius/pars horizontalis							
n	6	8	9	8	7	6	0
Mean±SD	61.7±93.5	96.3±114.3	81.7±109.8	53.1±48.0	77.1±72.3	80.0±59.7	-
Min	10	10	15	10	10	10	-
Median	25.0	60.0	20.0	35.0	50.0	80.0	-
Max	250	350	350	140	200	150	-
Splenius capitis							
n	30	29	27	26	23	17	3
Mean±SD	133.8±85.3	137.4±84.7	136.3±87.4	139.4±83.3	152.2±77.5	135.9±67.1	150.0±0.0
Min	20	30	20	30	40	40	150
Median	122.5	120.0	120.0	135.0	150.0	120.0	150.0
Max	300	300	300	300	300	260	150
Obliquus capitis superior							
n	1	1	1	1	1	1	0
Mean	15.0	20.0	20.0	20.0	20.0	20.0	-
Min	15	20	20	20	20	20	-
Median	15.0	20.0	20.0	20.0	20.0	20.0	-
Max	15	20	20	20	20	20	-
Obliquus capitis inferior							

Muscle group [a]	1st injection	2nd injection	3rd injection	4th injection	5th injection	6th injection	7th injection
n	10	13	13	12	12	6	0
Mean±SD	47.0±32.0	51.5±34.1	55.8±31.6	60.4±39.1	64.2±38.0	58.3±47.5	-
Min	10	10	10	10	10	20	-
Median	45.0	50.0	50.0	55.0	55.0	40.0	-
Max	100	100	100	130	130	130	-
Semispinalis capitis							
n	15	13	16	13	12	9	1
Mean±SD	56.0±30.2	61.5±29.0	69.7±42.1	65.8±29.6	77.5±31.7	71.1±34.4	50.0±(-)
Min	20	20	10	20	40	40	50
Median	50.0	50.0	50.0	50.0	80.0	60.0	50.0
Max	100	100	150	100	120	140	50
Levator scapulae							
n	17	21	20	15	15	12	2
Mean±SD	53.8±43.6	47.9±38.2	48.5±32.5	54.0±40.0	61.0±36.6	57.9±33.4	37.5±17.7
Min	10	10	15	15	20	20	25
Median	50.0	30.0	40.0	40.0	50.0	50.0	37.5
Max	150	150	120	150	150	140	50
Scalenus							
n	7	8	9	8	5	4	0
Mean±SD	60.7±49.7	60.6±53.6	54.4±59.8	67.5±73.2	110.0±112.2	92.5±99.8	-
Min	5	10	10	15	20	20	-
Median	50.0	50.0	40.0	55.0	60.0	55.0	-
Max	150	180	200	240	300	240	-
Platysma							
n	0	1	1	2	2	1	0
Mean±SD	-	80.0±(-)	80.0±(-)	65.0±21.2	65.0±21.2	80.0±(-)	-
Min	-	80	80	50	50	80	-
Median	-	80.0	80.0	65.0	65.0	80.0	-
Max	-	80	80	80	80	80	-
Paraspinal muscles							
n	3	3	3	2	3	2	1
Mean±SD	246.7±205.3	243.3±210.8	241.7±213.6	360.0±84.9	273.3±161.7	310.0±155.6	250.0±(-)
Min	20	10	5	300	100	200	250
Median	300.0	300.0	300.0	360.0	300.0	310.0	250.0
Max	420	420	420	420	420	420	250

**Muscle group** [a]	**1st injection**	**2nd injection**	**3rd injection**	**4th injection**	**5th injection**	**6th injection**	**7th injection**
Other (semispinalis cervicis)							
n	3	3	3	3	3	2	1
Mean±SD	60.0±17.3	75.0±25.0	83.3±28.9	83.3±28.9	76.7±25.2	75.0±35.4	50.0±(-)
Min	50	50	50	50	50	50	50
Median	50.0	75.0	100.0	100.0	80.0	75.0	50.0
Max	80	100	100	100	100	100	50
Other (rhomboid major)							
n	0	0	1	1	1	1	0
Mean	-	-	20.0	20.0	20.0	20.0	-
Min	-	-	20	20	20	20	-
Median	-	-	20.0	20.0	20.0	20.0	-
Max	-	-	20	20	20	20	-

[a] Multiple entries possible.

Max, maximum; Min, minimum; SD, standard deviation.

Table S3. TWSTRS total score change from the 1st injection cycle baseline by visit and muscle group (Group A).

	**1st injection**			**2nd injection**		**3rd injection**		**4th injection**		**5th injection**		**6th injection**		**7th injection**	
**Muscle group [a]**	Week 4	Week 8	Week 12	Baseline	Week 4	Baseline	Week 4	Baseline	Week 4	Baseline	Week 4	Baseline	Week 4	Baseline	Week 4
Sternocleidomastoid															
n	20	19	3	20	20	19	19	17	17	15	15	10	10	1	1
Mean±SD	-8.38±8.6	-7.28±9.5	-12.42±10.8	-7.30±9.1	-12.15±9.6	-9.42±8.7	-15.76±10.4	-9.41±9.2	-14.21±10.3	-9.83±9.2	-13.30±9.4	-13.75±8.4	-16.73±8.9	-12.75±(-)	-12.50±(-)
Trapezius															
n	9	8	1	9	9	9	9	8	8	7	7	6	6	2	2
Mean±SD	-9.67±9.4	-8.06±10.4	-17.75±(-)	-9.14±10.3	-12.33±10.7	-12.31±9.6	-15.19 ±12.6	-12.13±10.4	-13.66±12.4	-11.32±11.8	-13.14±11.0	-14.08±9.5	-15.38±8.9	-16.38±5.1	-16.75±6.0
Trapezius/pars horizontalis															
n	5	4	1	5	5	5	5	4	4	3	3	2	2	1	1
Mean±SD	-9.85±8.1	-7.19±7.9	-17.75±(-)	-9.30±8.3	-13.85±7.6	-15.50±7.6	-19.65±10.7	-19.94±2.4	-25.31±5.1	-21.17±4.3	-22.83±3.3	-22.88±4.8	-24.13±5.8	-20.00±(-)	-21.00±(-)
Splenius capitis															
n	27	26	4	27	27	26	26	24	24	22	22	15	15	3	3
Mean±SD	-11.01±9.4	-8.18±8.9	-11.56±9.0	-7.69±8.2	-13.29±9.1	-10.36±8.1	-15.89±9.3	-11.04±8.3	-15.28±9.2	-11.49±8.4	-14.91±8.5	-14.43±7.8	-17.95±8.6	-19.33±6.3	-18.67±5.4
Obliquus capitis superior															
n	1	1	0	1	1	1	1	1	1	1	1	1	1	0	0
Mean	-13.25	-12.25	-	-12.25	-17.75	19.50	-25.25	-22.75	-26.00	-26.00	-26.00	-26.25	-28.25	-	-
Obliquus capitis inferior															
n	8	8	2	8	8	8	8	7	7	7	7	3	3	0	0
Mean±SD	-4.19±9.8	-3.31±8.0	-4.50±6.4	-1.19±4.0	-8.16±5.4	-6.63±4.9	-10.03±6.3	-5.39±5.7	-9.79±5.2	-6.43±3.8	-11.11±6.1	-7.33±3.4	-10.17±3.8	-	-
Semispinalis capitis															
n	12	11	3	12	12	12	12	11	11	10	10	7	7	2	2
Mean±SD	-12.06±9.2	-10.93±8.9	-8.92±8.9	-10.08±8.4	-15.02±8.3	-13.52±8.4	-19.02±10.1	-14.00±8.4	-15.91±10.5	-13.68±9.6	-14.73±10.1	-15.18±10.1	-18.89±10.2	-16.38±5.1	-16.75±6.0
Levator scapulae															
n	15	14	1	15	15	15	15	15	15	13	13	10	10	2	2
Mean±SD	-9.90±9.8	-6.59±7.5	-17.75±(-)	-7.33±7.8	-12.50±8.5	-10.55±7.1	-16.25±9.5	-10.85±7.7	-14.95±10.3	-11.21±7.7	-13.23±8.2	-12.55±6.8	-16.33±7.9	-19.00±8.8	-17.50±7.1

Muscle group [a]	1st injection			2nd injection		3rd injection		4th injection		5th injection		6th injection		7th injection	
	Week 4	Week 8	Week 12	Baseline	Week 4	Baseline	Week 4	Baseline	Week 4	Baseline	Week 4	Baseline	Week 4	Baseline	Week 4
Scalenufs															
n	7	6	1	7	7	7	7	6	6	4	4	3	3	0	0
Mean±SD	-11.46± 6.4	-10.75± 7.2	-17.75± (-)	-11.75± 7.1	-14.25± 7.7	-11.61± 8.2	-19.39± 8.4	-11.83± 7.6	-20.17± 9.0	-10.75± 4.2	-17.94± 5.7	-13.33± 4.4	-13.08± 2.9	-	-
Platysma															
n	0	0	0	0	0	0	0	0	0	0	0	0	0	0	0
Mean±SD	-	-	-	-	-	-	-	-	-	-	-	-	-	-	-
Paraspinal															
n	3	3	0	3	3	3	3	2	2	2	2	2	2	1	1
Mean±SD	-15.08± 14.0	-8.25± 6.7	-	-8.25± 6.7	-13.75± 11.4	-8.75± 6.3	-18.83± 12.7	-11.88± 5.5	-15.88± 9.7	-11.13± 9.0	-14.50± 7.8	-13.00± 12.4	-17.25± 10.3	-25.25	-22.50
Other (semispinalis cervicis)															
n	3	3	1	3	3	3	3	3	3	3	3	2	2	1	1
Mean±SD	-19.50± 9.3	-19.08± 10.7	-9.00± (-)	-14.75± 11.5	-20.67± 12.0	-18.00± 11.2	-17.92± 11.2	-17.67± 9.8	-17.75± 9.7	-17.17± 12.0	-19.25± 10.9	-19.88± 14.3	-20.25± 14.8	-12.75	-12.50
Other (rhomboid major)															
n	0	0	0	0	0	0	0	0	0	0	0	0	0	0	0
Mean±SD	-	-	-	-	-	-	-	-	-	-	-	-	-	-	-

If the patient transferred to the 2nd injection cycle at the 1st injection Week 8 visit, and the visit matched the time window of the 1st injection cycle Week 8, the data were also treated as that of the 1st injection cycle Week 8 visit. The same applies for the 1st injection cycle Week 12 visit.

[a] For the 1st injection cycle, the injected muscle of the 1st injection cycle baseline was considered. For the 2nd and later injection cycles, the injected muscle of the most recent injection cycle baseline was considered.

SD, standard deviation; TWSTRS, Toronto Western Spasmodic Torticollis Rating Scale.

Table S4. CDIP-58 change from the 1st injection cycle baseline by visit (full analysis set).

Study visit	Group A		Group B (exploratory cohort)	
	n	Mean±SD	n	Mean±SD
1st injection cycle, Week 4	26	-7.07±11.5	3	-8.77±10.1
1st injection cycle, Week 8	26	-3.03±14.7	3	-6.90±3.7
2nd injection cycle, baseline	27	-3.82±15.1	3	-15.67±15.3
2nd injection cycle, Week 4	27	-5.86±18.4	3	-19.40±10.0
4th injection cycle, baseline	24	-7.59±18.6	3	-17.53±15.4
4th injection cycle, Week 4	24	-8.33±17.2	3	-11.93±12.5
6th injection cycle, Baseline	15	-4.19±20.5	2	-13.55±6.4
6th injection cycle, Week 4	15	-8.56±22.7	2	-12.90±7.9

Score = 25 * [Sum of the scores of the questions / Number of the questions - 1] (range from 0 to 100).

If the patient transferred to the 2nd injection cycle at the 1st injection Week 8 visit, and the visit matched the time window of the 1st injection cycle Week 8, the data were also treated as that of the 1st injection cycle Week 8 visit.

CDIP-58, Cervical Dystonia Impact Profile-58; SD, standard deviation.

Table S5. PEGR score trends across injection cycles by treatment group (full analysis set).

	Group A						Group B (exploratory cohort)	
	Total		Botulinum toxin–naïve		Previous botulinum toxin treatment		Previous botulinum toxin treatment	
**Study Visit**	**n**	**Mean±SD**	**n**	**Mean±SD**	**n**	**Mean±SD**	**n**	**Mean±SD**
1st injection cycle [a]	27	0.7±1.29	7	1.1±0.38	20	0.6±1.47	3	1.3±0.58
2nd injection cycle [b]	27	0.6±0.93	7	0.9±0.38	20	0.6±1.05	3	1.7±0.58
3rd injection cycle [b]	25	1.0±1.10	6	1.0±0.89	19	0.9±1.18	3	0.7±1.53
4th injection cycle [b]	24	0.8±1.33	6	1.0±1.10	18	0.7±1.41	3	1.7±0.58
5th injection cycle [b]	22	1.3±1.20	5	1.8±0.45	17	1.1±1.32	3	1.3±0.58
6th injection cycle [b]	15	1.3±1.39	5	1.6±0.89	10	1.1±1.60	2	1.5±0.71
7th injection cycle [b]	3	1.0±0.00	0	-	3	1.0±0.00	-	-

[a] 1st injection Week 20 or 2nd injection baseline.

[b] (n+1)th injection baseline (n=2-6).

PEGR, Patient Evaluation of Global Response; SD, standard deviation.

Table S6. Change in subscale D of the Modified Tsui Scale from the 1st injection baseline in patients with onset of head tremor at each injection cycle by visit (full analysis set).

	Patients with onset of head tremor at 1st injection baseline									
	Group A						Group B (exploratory cohort)		Total	
	Total		Botulinum toxin–naïve		Previous botulinum toxin treatment		Previous botulinum toxin treatment			
**Study visit**	**n**	**Mean±SD**	**n**	**Mean±SD**	**n**	**Mean±SD**	**n**	**Mean±SD**	**n**	**Mean±SD**
1st injection Week 4	16	-0.4±0.6	2	-1.0±0.0	14	-0.3±0.6	1	-3.0	17	-0.5±0.9
1st injection Week 8	16	-0.4±1.0	2	0.0±1.4	14	-0.5±0.9	1	-3.0	17	-0.6±1.1
1st injection Week 12	1	0.0	0	-	1	0.0	0	-	1	0.0
2nd injection Baseline	16	-0.4±1.0	2	0.0±1.4	14	-0.5±0.9	1	-3.0	17	-0.6±1.1
2nd injection Week 4	16	-0.7±0.9	2	-0.5±0.7	14	-0.7±0.9	1	-3.0	17	-0.8±1.0
3rd injection Baseline	16	-0.7±0.9	2	-0.5±0.7	14	-0.7±0.9	1	-3.0	17	-0.8±1.0
3rd injection Week 4	16	-0.8±0.9	2	-0.5±0.7	14	-0.8±0.9	1	-3.0	17	-0.9±1.0
4th injection Baseline	14	-0.7±1.0	2	-0.5±0.7	12	-0.8±1.1	1	-3.0	15	-0.9±1.1
4th injection Week 4	14	-0.7±0.9	2	-0.5±0.7	12	-0.8±1.0	1	-3.0	15	-0.9±1.1
5th injection Baseline	14	-0.6±0.9	2	-0.5±0.7	12	-0.7±1.0	1	-3.0	15	-0.8±1.1
5th injection Week 4	14	-0.7±0.9	2	-0.5±0.7	12	-0.8±1.0	1	-3.0	15	-0.9±1.1
6th injection Baseline	10	-1.0±0.9	2	-0.5±0.7	8	-1.1±1.0	1	-3.0	11	-1.2±1.1
6th injection Week 4	10	-0.9±1.0	2	-0.5±0.7	8	-1.0±1.1	1	-4.0	11	-1.2±1.3
7th injection Baseline	3	-0.7±0.6	0	-	3	-0.7±0.6	-	-	3	-0.7±0.6
7th injection Week 4	3	-0.7±0.6	0	-	3	-0.7±0.6	-	-	3	-0.7±0.6

SD, standard deviation.

Table S7. Related AEs by SOC and PT (safety analysis set).

SOC (PT)	Overall			1st injection cycle			2nd injection cycle			3rd injection cycle			4th injection cycle			5th injection cycle			6th injection cycle			7th injection cycle		
	Group A (n=27)	Group B (exploratory cohort) (n=3)	Total (N=30)	Group A (n=27)	Group B (exploratory cohort) (n=3)	Total (N=30)	Group A (n=27)	Group B (exploratory cohort) (n=3)	Total (N=30)	Group A (n=26)	Group B (exploratory cohort) (n=3)	Total (N=29)	Group A (n=24)	Group B (exploratory cohort) (n=3)	Total (N=27)	Group A (n=22)	Group B (exploratory cohort) (n=3)	Total (N=25)	Group A (n=15)	Group B (exploratory cohort) (n=2)	Total (N=17)	Group A (n=3)	Group B (exploratory cohort) (n=0)	Total (N=3)
Total	13 (48.1)	1 (33.3)	14 (46.7)	8 (29.6)	0 (0.0)	8 (26.7)	4 (14.8)	0 (0.0)	4 (13.3)	6 (23.1)	0 (0.0)	6 (20.7)	0 (0.0)	0 (0.0)	0 (0.0)	1 (4.5)	1 (33.3)	2 (8.0)	3 (20.0)	0 (0.0)	3 (17.6)	0 (0.0)	-	0 (0.0)
Nervous system disorders																								
Dizziness	1 (3.7)	0 (0.0)	1 (3.3)	1 (3.7)	0 (0.0)	1 (3.3)	0 (0.0)	0 (0.0)	0 (0.0)	0 (0.0)	0 (0.0)	0 (0.0)	0 (0.0)	0 (0.0)	0 (0.0)	0 (0.0)	0 (0.0)	0 (0.0)	0 (0.0)	0 (0.0)	0 (0.0)	0 (0.0)	-	0 (0.0)
Gastrointestinal disorders																								
Dysphagia	9 (33.3)	1 (33.3)	10 (33.3)	5 (18.5)	0 (0.0)	5 (16.7)	4 (14.8)	0 (0.0)	4 (13.3)	3 (11.5)	0 (0.0)	3 (10.3)	0 (0.0)	0 (0.0)	0 (0.0)	1 (4.5)	1 (33.3)	2 (8.0)	1 (6.7)	0 (0.0)	1 (5.9)	0 (0.0)	-	0 (0.0)
Musculoskeletal and connective tissue disorders																								
Back pain	1 (3.7)	0 (0.0)	1 (3.3)	0 (0.0)	0 (0.0)	0 (0.0)	1 (3.7)	0 (0.0)	1 (3.3)	0 (0.0)	0 (0.0)	0 (0.0)	0 (0.0)	0 (0.0)	0 (0.0)	0 (0.0)	0 (0.0)	0 (0.0)	0 (0.0)	0 (0.0)	0 (0.0)	0 (0.0)	-	0 (0.0)
Muscle atrophy	1 (3.7)	0 (0.0)	1 (3.3)	0 (0.0)	0 (0.0)	0 (0.0)	0 (0.0)	0 (0.0)	0 (0.0)	1 (3.8)	0 (0.0)	1 (3.4)	0 (0.0)	0 (0.0)	0 (0.0)	0 (0.0)	0 (0.0)	0 (0.0)	0 (0.0)	0 (0.0)	0 (0.0)	0 (0.0)	-	0 (0.0)
Muscle spasms	1 (3.7)	0 (0.0)	1 (3.3)	1 (3.7)	0 (0.0)	1 (3.3)	0 (0.0)	0 (0.0)	0 (0.0)	0 (0.0)	0 (0.0)	0 (0.0)	0 (0.0)	0 (0.0)	0 (0.0)	0 (0.0)	0 (0.0)	0 (0.0)	0 (0.0)	0 (0.0)	0 (0.0)	0 (0.0)	-	0 (0.0)
Muscular weakness	6 (22.2)	0 (0.0)	6 (20.0)	3 (11.1)	0 (0.0)	3 (10.0)	2 (7.4)	0 (0.0)	2 (6.7)	2 (7.7)	0 (0.0)	2 (6.9)	0 (0.0)	0 (0.0)	0 (0.0)	0 (0.0)	0 (0.0)	0 (0.0)	2 (13.3)	0 (0.0)	2 (11.8)	0 (0.0)	-	0 (0.0)
Myalgia	1 (3.7)	0 (0.0)	1 (3.3)	0 (0.0)	0 (0.0)	0 (0.0)	1 (3.7)	0 (0.0)	1 (3.3)	0 (0.0)	0 (0.0)	0 (0.0)	0 (0.0)	0 (0.0)	0 (0.0)	0 (0.0)	0 (0.0)	0 (0.0)	0 (0.0)	0 (0.0)	0 (0.0)	0 (0.0)	-	0 (0.0)
General disorders and administration site conditions																								
Feeling abnormal	1 (3.7)	0 (0.0)	1 (3.3)	1 (3.7)	0 (0.0)	1 (3.3)	0 (0.0)	0 (0.0)	0 (0.0)	0 (0.0)	0 (0.0)	0 (0.0)	0 (0.0)	0 (0.0)	0 (0.0)	0 (0.0)	0 (0.0)	0 (0.0)	0 (0.0)	0 (0.0)	0 (0.0)	0 (0.0)	-	0 (0.0)
Injection site pain	1 (3.7)	0 (0.0)	1 (3.3)	0 (0.0)	0 (0.0)	0 (0.0)	1 (3.7)	0 (0.0)	1 (3.3)	1 (3.8)	0 (0.0)	1 (3.4)	0 (0.0)	0 (0.0)	0 (0.0)	0 (0.0)	0 (0.0)	0 (0.0)	0 (0.0)	0 (0.0)	0 (0.0)	0 (0.0)	-	0 (0.0)
Injection site warmth	1 (3.7)	0 (0.0)	1 (3.3)	1 (3.7)	0 (0.0)	1 (3.3)	0 (0.0)	0 (0.0)	0 (0.0)	0 (0.0)	0 (0.0)	0 (0.0)	0 (0.0)	0 (0.0)	0 (0.0)	0 (0.0)	0 (0.0)	0 (0.0)	0 (0.0)	0 (0.0)	0 (0.0)	0 (0.0)	-	0 (0.0)

Results are presented as n (%).

Patients with one or more AEs within a level of MedDRA term are counted only once in that level.

Percentages are based on the number of patients in the safety set for each group.

AE, adverse event; PT, preferred term; SOC, system organ class.

Table S8. AEs that occurred in ≥2 patients in the pooled population (safety analysis set).

SOC (PT)	Overall			1st injection cycle			2nd injection cycle			3rd injection cycle			4th injection cycle			5th injection cycle			6th injection cycle			7th injection cycle		
	Group A (n=27)	Group B (exploratory cohort) (n=3)	Total (N=30)	Group A (n=27)	Group B (exploratory cohort) (n=3)	Total (N=30)	Group A (n=27)	Group B (exploratory cohort) (n=3)	Total (N=30)	Group A (n=26)	Group B (exploratory cohort) (n=3)	Total (N=29)	Group A (n=24)	Group B (exploratory cohort) (n=3)	Total (N=27)	Group A (n=22)	Group B (exploratory cohort) (n=3)	Total (N=25)	Group A (n=15)	Group B (exploratory cohort) (n=2)	Total (N=17)	Group A (n=3)	Group B (exploratory cohort) (n=0)	Total (N=3)
Total	18 (66.7)	3 (100.0)	21 (70.0)	7 (25.9)	1 (33.3)	8 (26.7)	9 (33.3)	1 (33.3)	10 (33.3)	7 (26.9)	0 (0.0)	7 (24.1)	3 (12.5)	0 (0.0)	3 (11.1)	2 (9.1)	2 (66.7)	4 (16.0)	7 (46.7)	0 (0.0)	7 (41.2)	0 (0.0)	0 (-)	0 (0.0)
Infections and infestations																								
Influenza	1 (3.7)	1 (33.3)	2 (6.7)	0 (0.0)	0 (0.0)	0 (0.0)	0 (0.0)	1 (33.3)	1 (3.3)	0 (0.0)	0 (0.0)	0 (0.0)	1 (4.2)	0 (0.0)	1 (3.7)	0 (0.0)	0 (0.0)	0 (0.0)	0 (0.0)	0 (0.0)	0 (0.0)	0 (0.0)	0 (-)	0 (0.0)
Nasopharyngitis	6 (22.2)	1 (33.3)	7 (23.3)	0 (0.0)	0 (0.0)	0 (0.0)	2 (7.4)	0 (0.0)	2 (6.7)	2 (7.7)	0 (0.0)	2 (6.9)	2 (8.3)	0 (0.0)	2 (7.4)	1 (4.5)	1 (33.3)	2 (8.0)	1 (6.7)	0 (0.0)	1 (5.9)	0 (0.0)	0 (-)	0 (0.0)
COVID-19	3 (11.1)	0 (0.0)	3 (10.0)	0 (0.0)	0 (0.0)	0 (0.0)	1 (3.7)	0 (0.0)	1 (3.3)	0 (0.0)	0 (0.0)	0 (0.0)	0 (0.0)	0 (0.0)	0 (0.0)	0 (0.0)	0 (0.0)	0 (0.0)	2 (13.3)	0 (0.0)	2 (11.8)	0 (0.0)	0 (-)	0 (0.0)
Gastrointestinal disorders																								
Dental caries	1 (3.7)	1 (33.3)	2 (6.7)	0 (0.0)	1 (33.3)	1 (3.3)	1 (3.7)	0 (0.0)	1 (3.3)	0 (0.0)	0 (0.0)	0 (0.0)	0 (0.0)	0 (0.0)	0 (0.0)	0 (0.0)	0 (0.0)	0 (0.0)	1 (6.7)	0 (0.0)	1 (5.9)	0 (0.0)	0 (-)	0 (0.0)
Dysphagia	9 (33.3)	1 (33.3)	10 (33.3)	5 (18.5)	0 (0.0)	5 (16.7)	4 (14.8)	0 (0.0)	4 (13.3)	3 (11.5)	0 (0.0)	3 (10.3)	0 (0.0)	0 (0.0)	0 (0.0)	1 (4.5)	1 (33.3)	2 (8.0)	1 (6.7)	0 (0.0)	1 (5.9)	0 (0.0)	0 (-)	0 (0.0)
Musculoskeletal and connective tissue disorders																								
Muscular weakness	6 (22.2)	0 (0.0)	6 (20.0)	3 (11.1)	0 (0.0)	3 (10.0)	2 (7.4)	0 (0.0)	2 (6.7)	2 (7.7)	0 (0.0)	2 (6.9)	0 (0.0)	0 (0.0)	0 (0.0)	0 (0.0)	0 (0.0)	0 (0.0)	2 (13.3)	0 (0.0)	2 (11.8)	0 (0.0)	0 (-)	0 (0.0)
Injury, poisoning and procedural complications																								
Contusion	2 (7.4)	0 (0.0)	2 (6.7)	1 (3.7)	0 (0.0)	1 (3.3)	1 (3.7)	0 (0.0)	1 (3.3)	0 (0.0)	0 (0.0)	0 (0.0)	0 (0.0)	0 (0.0)	0 (0.0)	0 (0.0)	0 (0.0)	0 (0.0)	0 (0.0)	0 (0.0)	0 (0.0)	0 (0.0)	0 (-)	0 (0.0)

Results are presented as n (%).

Patients with one or more AEs within a level of MedDRA term were counted only once in that level. Percentages are based on the number of patients in the safety set for each group.

MedDRA version 27.1 was used for coding AEs.

AE, adverse event; COVID-19, coronavirus disease 2019; MedDRA, Medical Dictionary for Regulatory Activities; PT, preferred term; SOC, system organ class.

Table S9. AEs by dose of incobotulinumtoxinA.

	Group A						Group B (exploratory cohort)						Total					
	<120 U (n=1)	≥120–<240 U (n=9)	≥240–<300 U (n=9)	≥300–<400 U (n=6)	≥400–<500 U (n=11)	500 U (n=14)	<120 U (n=0)	≥120–<240 U (n=1)	≥240–<300 U (n=0)	≥300–<400 U (n=1)	≥400–<500 U (n=1)	500 U (n=2)	<120 U (n=1)	≥120–<240 U (n=10)	≥240–<300 U (n=9)	≥300–<400 U (n=7)	≥400–<500 U (n=12)	500 U (n=16)
Total	1 (100.0)	5 (55.6)	4 (44.4)	2 (33.3)	7 (63.6)	9 (64.3)	0 (-)	1 (100.0)	0 (-)	1 (100.0)	1 (100.0)	2 (100.0)	1 (100.0)	6 (60.0)	4 (44.4)	3 (42.9)	8 (66.7)	11 (68.8)
Infections and infestations																		
Folliculitis	0 (0.0)	0 (0.0)	0 (0.0)	0 (0.0)	0 (0.0)	1 (7.1)	0 (-)	0 (0.0)	0 (-)	0 (0.0)	0 (0.0)	0 (0.0)	0 (0.0)	0 (0.0)	0 (0.0)	0 (0.0)	0 (0.0)	1 (6.3)
Gastroenteritis	0 (0.0)	0 (0.0)	0 (0.0)	0 (0.0)	0 (0.0)	1 (7.1)	0 (-)	0 (0.0)	0 (-)	0 (0.0)	0 (0.0)	0 (0.0)	0 (0.0)	0 (0.0)	0 (0.0)	0 (0.0)	0 (0.0)	1 (6.3)
Influenza	0 (0.0)	0 (0.0)	0 (0.0)	0 (0.0)	1 (9.1)	0 (0.0)	0 (-)	0 (0.0)	0 (-)	0 (0.0)	0 (0.0)	1 (50.0)	0 (0.0)	0 (0.0)	0 (0.0)	0 (0.0)	1 (8.3)	1 (6.3)
Nasopharyngitis	0 (0.0)	1 (11.1)	0 (0.0)	0 (0.0)	2 (18.2)	3 (21.4)	0 (-)	1 (100.0)	0 (-)	0 (0.0)	0 (0.0)	0 (0.0)	0 (0.0)	2 (20.0)	0 (0.0)	0 (0.0)	2 (16.7)	3 (18.8)
Sinusitis	0 (0.0)	0 (0.0)	0 (0.0)	0 (0.0)	0 (0.0)	1 (7.1)	0 (-)	0 (0.0)	0 (-)	0 (0.0)	0 (0.0)	0 (0.0)	0 (0.0)	0 (0.0)	0 (0.0)	0 (0.0)	0 (0.0)	1 (6.3)
COVID-19	0 (0.0)	0 (0.0)	1 (11.1)	0 (0.0)	0 (0.0)	2 (14.3)	0 (-)	0 (0.0)	0 (-)	0 (0.0)	0 (0.0)	0 (0.0)	0 (0.0)	0 (0.0)	1 (11.1)	0 (0.0)	0 (0.0)	2 (12.5)
Suspected COVID-19	0 (0.0)	0 (0.0)	0 (0.0)	0 (0.0)	0 (0.0)	1 (7.1)	0 (-)	0 (0.0)	0 (-)	0 (0.0)	0 (0.0)	0 (0.0)	0 (0.0)	0 (0.0)	0 (0.0)	0 (0.0)	0 (0.0)	1 (6.3)
Endocrine disorders																		
Hyperthyroidism	0 (0.0)	1 (11.1)	0 (0.0)	0 (0.0)	0 (0.0)	0 (0.0)	0 (-)	0 (0.0)	0 (-)	0 (0.0)	0 (0.0)	0 (0.0)	0 (0.0)	1 (10.0)	0 (0.0)	0 (0.0)	0 (0.0)	0 (0.0)
Metabolism and nutrition disorders																		
Dyslipidemia	0 (0.0)	1 (11.1)	0 (0.0)	0 (0.0)	0 (0.0)	0 (0.0)	0 (-)	0 (0.0)	0 (-)	0 (0.0)	0 (0.0)	0 (0.0)	0 (0.0)	1 (10.0)	0 (0.0)	0 (0.0)	0 (0.0)	0 (0.0)
Nervous system disorders																		
Dizziness	0 (0.0)	0 (0.0)	0 (0.0)	1 (16.7)	0 (0.0)	0 (0.0)	0 (-)	0 (0.0)	0 (-)	0 (0.0)	0 (0.0)	0 (0.0)	0 (0.0)	0 (0.0)	0 (0.0)	1 (14.3)	0 (0.0)	0 (0.0)
Head discomfort	0 (0.0)	0 (0.0)	0 (0.0)	1 (16.7)	0 (0.0)	0 (0.0)	0 (-)	0 (0.0)	0 (-)	0 (0.0)	0 (0.0)	0 (0.0)	0 (0.0)	0 (0.0)	0 (0.0)	1 (14.3)	0 (0.0)	0 (0.0)
Vascular disorders																		
Hypertension	1 (100.0)	0 (0.0)	0 (0.0)	0 (0.0)	0 (0.0)	0 (0.0)	0 (-)	0 (0.0)	0 (-)	0 (0.0)	0 (0.0)	0 (0.0)	1 (100.0)	0 (0.0)	0 (0.0)	0 (0.0)	0 (0.0)	0 (0.0)
Gastrointestinal disorders																		
Cheilitis	0 (0.0)	0 (0.0)	0 (0.0)	0 (0.0)	0 (0.0)	0 (0.0)	0 (-)	0 (0.0)	0 (-)	1 (100.0)	0 (0.0)	0 (0.0)	0 (0.0)	0 (0.0)	0 (0.0)	1 (14.3)	0 (0.0)	0 (0.0)
Constipation	0 (0.0)	1 (11.1)	0 (0.0)	0 (0.0)	0 (0.0)	0 (0.0)	0 (-)	0 (0.0)	0 (-)	0 (0.0)	0 (0.0)	0 (0.0)	0 (0.0)	1 (10.0)	0 (0.0)	0 (0.0)	0 (0.0)	0 (0.0)
Dental caries	0 (0.0)	0 (0.0)	0 (0.0)	0 (0.0)	0 (0.0)	1 (7.1)	0 (-)	0 (0.0)	0 (-)	0 (0.0)	1 (100.0)	0 (0.0)	0 (0.0)	0 (0.0)	0 (0.0)	0 (0.0)	1 (8.3)	1 (6.3)
Dysphagia	0 (0.0)	3 (33.3)	3 (33.3)	1 (16.7)	3 (27.3)	2 (14.3)	0 (-)	0 (0.0)	0 (-)	0 (0.0)	0 (0.0)	1 (50.0)	0 (0.0)	3 (30.0)	3 (33.3)	1 (14.3)	3 (25.0)	3 (18.8)
Glossitis	0 (0.0)	0 (0.0)	0 (0.0)	1 (16.7)	0 (0.0)	0 (0.0)	0 (-)	0 (0.0)	0 (-)	0 (0.0)	0 (0.0)	0 (0.0)	0 (0.0)	0 (0.0)	0 (0.0)	1 (14.3)	0 (0.0)	0 (0.0)
Skin and subcutaneous tissue disorders																		
Acne	0 (0.0)	0 (0.0)	0 (0.0)	0 (0.0)	0 (0.0)	1 (7.1)	0 (-)	0 (0.0)	0 (-)	0 (0.0)	0 (0.0)	0 (0.0)	0 (0.0)	0 (0.0)	0 (0.0)	0 (0.0)	0 (0.0)	1 (6.3)
Eczema	0 (0.0)	0 (0.0)	0 (0.0)	0 (0.0)	0 (0.0)	1 (7.1)	0 (-)	0 (0.0)	0 (-)	0 (0.0)	0 (0.0)	0 (0.0)	0 (0.0)	0 (0.0)	0 (0.0)	0 (0.0)	0 (0.0)	1 (6.3)
Rash	0 (0.0)	1 (11.1)	0 (0.0)	0 (0.0)	0 (0.0)	0 (0.0)	0 (-)	0 (0.0)	0 (-)	0 (0.0)	0 (0.0)	0 (0.0)	0 (0.0)	1 (10.0)	0 (0.0)	0 (0.0)	0 (0.0)	0 (0.0)
Musculoskeletal and connective tissue disorders																		
Arthralgia	1 (100.0)	0 (0.0)	0 (0.0)	0 (0.0)	0 (0.0)	0 (0.0)	0 (-)	0 (0.0)	0 (-)	0 (0.0)	0 (0.0)	0 (0.0)	1 (100.0)	0 (0.0)	0 (0.0)	0 (0.0)	0 (0.0)	0 (0.0)
Back pain	0 (0.0)	0 (0.0)	0 (0.0)	1 (16.7)	0 (0.0)	0 (0.0)	0 (-)	0 (0.0)	0 (-)	0 (0.0)	0 (0.0)	0 (0.0)	0 (0.0)	0 (0.0)	0 (0.0)	1 (14.3)	0 (0.0)	0 (0.0)
Muscle atrophy	0 (0.0)	0 (0.0)	1 (11.1)	0 (0.0)	0 (0.0)	0 (0.0)	0 (-)	0 (0.0)	0 (-)	0 (0.0)	0 (0.0)	0 (0.0)	0 (0.0)	0 (0.0)	1 (11.1)	0 (0.0)	0 (0.0)	0 (0.0)
Muscle Spasms	0 (0.0)	0 (0.0)	0 (0.0)	0 (0.0)	0 (0.0)	1 (7.1)	0 (-)	0 (0.0)	0 (-)	0 (0.0)	0 (0.0)	0 (0.0)	0 (0.0)	0 (0.0)	0 (0.0)	0 (0.0)	0 (0.0)	1 (6.3)
Muscular weakness	1 (100.0)	1 (11.1)	1 (11.1)	1 (16.7)	1 (9.1)	2 (14.3)	0 (-)	0 (0.0)	0 (-)	0 (0.0)	0 (0.0)	0 (0.0)	1 (100.0)	1 (10.0)	1 (11.1)	1 (14.3)	1 (8.3)	2 (12.5)
Myalgia	0 (0.0)	0 (0.0)	0 (0.0)	1 (16.7)	0 (0.0)	0 (0.0)	0 (-)	0 (0.0)	0 (-)	0 (0.0)	0 (0.0)	0 (0.0)	0 (0.0)	0 (0.0)	0 (0.0)	1 (14.3)	0 (0.0)	0 (0.0)

	Group A						Group B (exploratory cohort)						Total					
	**<120 U (n=1)**	**≥120–<240 U (n=9)**	**≥240–<300 U (n=9)**	**≥300–<400 U (n=6)**	**≥400–<500 U (n=11)**	**500 U (n=14)**	**<120 U (n=0)**	**≥120–<240 U (n=1)**	**≥240–<300 U (n=0)**	**≥300–<400 U (n=1)**	**≥400–<500 U (n=1)**	**500 U (n=2)**	**<120 U (n=1)**	**≥120–<240 U (n=10)**	**≥240–<300 U (n=9)**	**≥300–<400 U (n=7)**	**≥400–<500 U (n=12)**	**500 U (n=16)**
Neck pain	1 (100.0)	0 (0.0)	0 (0.0)	0 (0.0)	0 (0.0)	0 (0.0)	0 (-)	0 (0.0)	0 (-)	0 (0.0)	0 (0.0)	0 (0.0)	1 (100.0)	0 (0.0)	0 (0.0)	0 (0.0)	0 (0.0)	0 (0.0)
Spinal osteoarthritis	0 (0.0)	0 (0.0)	1 (11.1)	0 (0.0)	0 (0.0)	0 (0.0)	0 (-)	0 (0.0)	0 (-)	0 (0.0)	0 (0.0)	0 (0.0)	0 (0.0)	0 (0.0)	1 (11.1)	0 (0.0)	0 (0.0)	0 (0.0)
General disorders and administration site conditions																		
Feeling abnormal	0 (0.0)	0 (0.0)	0 (0.0)	1 (16.7)	0 (0.0)	0 (0.0)	0 (-)	0 (0.0)	0 (-)	0 (0.0)	0 (0.0)	0 (0.0)	0 (0.0)	0 (0.0)	0 (0.0)	1 (14.3)	0 (0.0)	0 (0.0)
Injection site pain	0 (0.0)	0 (0.0)	1 (11.1)	1 (16.7)	0 (0.0)	0 (0.0)	0 (-)	0 (0.0)	0 (-)	0 (0.0)	0 (0.0)	0 (0.0)	0 (0.0)	0 (0.0)	1 (11.1)	1 (14.3)	0 (0.0)	0 (0.0)
Injection site warmth	0 (0.0)	0 (0.0)	0 (0.0)	0 (0.0)	0 (0.0)	1 (7.1)	0 (-)	0 (0.0)	0 (-)	0 (0.0)	0 (0.0)	0 (0.0)	0 (0.0)	0 (0.0)	0 (0.0)	0 (0.0)	0 (0.0)	1 (6.3)
Pyrexia	0 (0.0)	0 (0.0)	0 (0.0)	0 (0.0)	0 (0.0)	1 (7.1)	0 (-)	0 (0.0)	0 (-)	0 (0.0)	0 (0.0)	0 (0.0)	0 (0.0)	0 (0.0)	0 (0.0)	0 (0.0)	0 (0.0)	1 (6.3)
Investigations																		
Blood creatine phosphokinase increased	0 (0.0)	0 (0.0)	0 (0.0)	0 (0.0)	0 (0.0)	1 (7.1)	0 (-)	0 (0.0)	0 (-)	0 (0.0)	0 (0.0)	0 (0.0)	0 (0.0)	0 (0.0)	0 (0.0)	0 (0.0)	0 (0.0)	1 (6.3)
Gamma-glutamyl transferase increased	0 (0.0)	0 (0.0)	0 (0.0)	0 (0.0)	0 (0.0)	1 (7.1)	0 (-)	0 (0.0)	0 (-)	0 (0.0)	0 (0.0)	0 (0.0)	0 (0.0)	0 (0.0)	0 (0.0)	0 (0.0)	0 (0.0)	1 (6.3)
Hepatic enzyme increased	0 (0.0)	0 (0.0)	0 (0.0)	0 (0.0)	0 (0.0)	1 (7.1)	0 (-)	0 (0.0)	0 (-)	0 (0.0)	0 (0.0)	0 (0.0)	0 (0.0)	0 (0.0)	0 (0.0)	0 (0.0)	0 (0.0)	1 (6.3)
Injury, poisoning and procedural complications																		
Contusion	0 (0.0)	2 (22.2)	0 (0.0)	0 (0.0)	0 (0.0)	0 (0.0)	0 (-)	0 (0.0)	0 (-)	0 (0.0)	0 (0.0)	0 (0.0)	0 (0.0)	2 (20.0)	0 (0.0)	0 (0.0)	0 (0.0)	0 (0.0)
Heat illness	0 (0.0)	0 (0.0)	0 (0.0)	0 (0.0)	0 (0.0)	1 (7.1)	0 (-)	0 (0.0)	0 (-)	0 (0.0)	0 (0.0)	0 (0.0)	0 (0.0)	0 (0.0)	0 (0.0)	0 (0.0)	0 (0.0)	1 (6.3)

Results are presented as n (%).

AE, adverse event; COVID-19, coronavirus disease 2019.

Table S10. AEs during treatment with incobotulinumtoxinA at each stage of administration (safety analysis set).

	**Overall**			**1st injection cycle**			**2nd injection cycle**			**3rd injection cycle**			**4th injection cycle**			**5th injection cycle**			**6th injection cycle**			**7th injection cycle**		
	**Group A (N=27) n (%)**	**Group B (exploratory cohort) (N=3) n (%)**	**Total (N=30) n (%)**	**Group A (N=27) n (%)**	**Group B (exploratory cohort) (N=3) n (%)**	**Total (N=30) n (%)**	**Group A (N=27) n (%)**	**Group B (exploratory cohort) (N=3) n (%)**	**Total (N=30) n (%)**	**Group A (N=26) n (%)**	**Group B (exploratory cohort) (N=3) n (%)**	**Total (N=29) n (%)**	**Group A (N=24) n (%)**	**Group B (exploratory cohort) (N=3) n (%)**	**Total (N=27) n (%)**	**Group A (N=22) n (%)**	**Group B (exploratory cohort) (N=3) n (%)**	**Total (N=25) n (%)**	**Group A (N=15) n (%)**	**Group B (exploratory cohort) (N=2) n (%)**	**Total (N=17) n (%)**	**Group A (N=3) n (%)**	**Group B (exploratory cohort) (N=0) n (%)**	**Total (N=3) n (%)**
Any AE	21 (77.8)	3 (100.0)	24 (80.0)	9 (33.3)	1 (33.3)	10 (33.3)	13 (48.1)	1 (33.3)	14 (46.7)	12 (46.2)	0 (0.0)	12 (41.4)	4 (16.7)	0 (0.0)	4 (14.8)	4 (18.2)	2 (66.7)	6 (24.0)	7 (46.7)	1 (50.0)	8 (47.1)	2 (66.7)	0 (-)	2 (66.7)
Any related AE	13 (48.1)	1 (33.3)	14 (46.7)	8 (29.6)	0 (0.0)	8 (26.7)	4 (14.8)	0 (0.0)	4 (13.3)	6 (23.1)	0 (0.0)	6 (20.7)	0 (0.0)	0 (0.0)	0 (0.0)	1 (4.5)	1 (33.3)	2 (8.0)	3 (20.0)	0 (0.0)	3 (17.6)	0 (0.0)	0 (-)	0 (0.0)
Leading to death																								
AE	0 (0.0)	0 (0.0)	0 (0.0)	0 (0.0)	0 (0.0)	0 (0.0)	0 (0.0)	0 (0.0)	0 (0.0)	0 (0.0)	0 (0.0)	0 (0.0)	0 (0.0)	0 (0.0)	0 (0.0)	0 (0.0)	0 (0.0)	0 (0.0)	0 (0.0)	0 (0.0)	0 (0.0)	0 (0.0)	0 (-)	0 (0.0)
Related AE	0 (0.0)	0 (0.0)	0 (0.0)	0 (0.0)	0 (0.0)	0 (0.0)	0 (0.0)	0 (0.0)	0 (0.0)	0 (0.0)	0 (0.0)	0 (0.0)	0 (0.0)	0 (0.0)	0 (0.0)	0 (0.0)	0 (0.0)	0 (0.0)	0 (0.0)	0 (0.0)	0 (0.0)	0 (0.0)	0 (-)	0 (0.0)
Serious																								
AE	1 (3.7)	0 (0.0)	1 (3.3)	1 (3.7)	0 (0.0)	1 (3.3)	0 (0.0)	0 (0.0)	0 (0.0)	0 (0.0)	0 (0.0)	0 (0.0)	0 (0.0)	0 (0.0)	0 (0.0)	0 (0.0)	0 (0.0)	0 (0.0)	0 (0.0)	0 (0.0)	0 (0.0)	0 (0.0)	0 (-)	0 (0.0)
Related AE	0 (0.0)	0 (0.0)	0 (0.0)	0 (0.0)	0 (0.0)	0 (0.0)	0 (0.0)	0 (0.0)	0 (0.0)	0 (0.0)	0 (0.0)	0 (0.0)	0 (0.0)	0 (0.0)	0 (0.0)	0 (0.0)	0 (0.0)	0 (0.0)	0 (0.0)	0 (0.0)	0 (0.0)	0 (0.0)	0 (-)	0 (0.0)
Leading to discontinuation of the study																								
AE	0 (0.0)	0 (0.0)	0 (0.0)	0 (0.0)	0 (0.0)	0 (0.0)	0 (0.0)	0 (0.0)	0 (0.0)	0 (0.0)	0 (0.0)	0 (0.0)	0 (0.0)	0 (0.0)	0 (0.0)	0 (0.0)	0 (0.0)	0 (0.0)	0 (0.0)	0 (0.0)	0 (0.0)	0 (0.0)	0 (-)	0 (0.0)
Related AE	0 (0.0)	0 (0.0)	0 (0.0)	0 (0.0)	0 (0.0)	0 (0.0)	0 (0.0)	0 (0.0)	0 (0.0)	0 (0.0)	0 (0.0)	0 (0.0)	0 (0.0)	0 (0.0)	0 (0.0)	0 (0.0)	0 (0.0)	0 (0.0)	0 (0.0)	0 (0.0)	0 (0.0)	0 (0.0)	0 (-)	0 (0.0)
Maximum severity																								
AE [a]																								
Mild	12 (44.4)	3 (100.0)	15 (50.0)	5 (18.5)	1 (33.3)	6 (20.0)	9 (33.3)	1 (33.3)	10 (33.3)	9 (34.6)	0 (0.0)	9 (31.0)	3 (12.5)	0 (0.0)	3 (11.1)	3 (13.6)	2 (66.7)	5 (20.0)	7 (46.7)	1 (50.0)	8 (47.1)	2 (66.7)	0 (-)	2 (66.7)
Moderate	9 (33.3)	0 (0.0)	9 (30.0)	4 (14.8)	0 (0.0)	4 (13.3)	4 (14.8)	0 (0.0)	4 (13.3)	3 (11.5)	0 (0.0)	3 (10.3)	1 (4.2)	0 (0.0)	1 (3.7)	1 (4.5)	0 (0.0)	1 (4.0)	0 (0.0)	0 (0.0)	0 (0.0)	0 (0.0)	0 (-)	0 (0.0)
Severe	0 (0.0)	0 (0.0)	0 (0.0)	0 (0.0)	0 (0.0)	0 (0.0)	0 (0.0)	0 (0.0)	0 (0.0)	0 (0.0)	0 (0.0)	0 (0.0)	0 (0.0)	0 (0.0)	0 (0.0)	0 (0.0)	0 (0.0)	0 (0.0)	0 (0.0)	0 (0.0)	0 (0.0)	0 (0.0)	0 (-)	0 (0.0)
Related AE [a]																								
Mild	7 (25.9)	1 (33.3)	8 (26.7)	4 (14.8)	0 (0.0)	4 (13.3)	2 (7.4)	0 (0.0)	2 (6.7)	4 (15.4)	0 (0.0)	4 (13.8)	0 (0.0)	0 (0.0)	0 (0.0)	1 (4.5)	1 (33.3)	2 (8.0)	3 (20.0)	0 (0.0)	3 (17.6)	0 (0.0)	0 (-)	0 (0.0)
Moderate	6 (22.2)	0 (0.0)	6 (20.0)	4 (14.8)	0 (0.0)	4 (13.3)	2 (7.4)	0 (0.0)	2 (6.7)	2 (7.7)	0 (0.0)	2 (6.9)	0 (0.0)	0 (0.0)	0 (0.0)	0 (0.0)	0 (0.0)	0 (0.0)	0 (0.0)	0 (0.0)	0 (0.0)	0 (0.0)	0 (-)	0 (0.0)
Severe	0 (0.0)	0 (0.0)	0 (0.0)	0 (0.0)	0 (0.0)	0 (0.0)	0 (0.0)	0 (0.0)	0 (0.0)	0 (0.0)	0 (0.0)	0 (0.0)	0 (0.0)	0 (0.0)	0 (0.0)	0 (0.0)	0 (0.0)	0 (0.0)	0 (0.0)	0 (0.0)	0 (0.0)	0 (0.0)	0 (-)	0 (0.0)

AEs are defined as any AE, regardless of relationship to the study drug. Percentages are based on the total number of patients in the safety set for each group.

[a] Patients with one or more AEs within a level of the MedDRA term are counted only once in that level using the most severe incident.

AE, adverse event; MedDRA, Medical Dictionary for Regulatory Activities.

Table S11. The details of the institutional review board approvals for all 15 participating institutions (Protocol number: NT 201C-301).

Name of the institutional review board	Name of the participating institution	Approval status	Date of approval
Nakamura Memorial Hospital Institutional Review Board	Nakamura Memorial Hospital	Approved	February 16, 2024
Yokohama Minoru Clinic Institutional Review Board	Jichi Medical University Station Brain Clinic	Approved	February 16, 2024
Chiba University Hospital Institutional Review Board	Chiba University Hospital	Approved	March 18, 2024
National Center of Neurology and Psychiatry Institutional Review Board	National Center of Neurology and Psychiatry	Approved	March 22, 2024
Adachikyousai Hospital Institutional Review Board	Tamagawa Clinic	Approved	February 22, 2024
Takeda Hospital Group Institutional Review Board	Yanaginobaba Takeda Clinic	Approved	March 28, 2024
The University of Osaka Hospital Institutional Review Board	The University of Osaka Hospital	Approved	April 16, 2024
Tokushima University Hospital Institutional Review Board	Tokushima University Hospital	Approved	March 26, 2024
Fukuoka University Hospital Institutional Review Board	Fukuoka University Hospital	Approved	February 28, 2024
Adachikyousai Hospital Institutional Review Board	Ookatsu Hospital	Approved	February 22, 2024
Adachikyousai Hospital Institutional Review Board	Yui Odawara Clinic	Approved	February 22, 2024
Nara Prefecture General Medical Center Institutional Review Board	Nara Prefecture General Medical Center	Approved	March 19, 2024
Adachikyousai Hospital Institutional Review Board	Osaka Neurological Institute	Approved	March 28, 2024
Juntendo University Hospital Institutional Review Board	Juntendo University Hospital	Approved	April 23, 2024
Kawasaki Medical School Hospital Institutional Review Board	Kawasaki Medical School Hospital	Approved	May 24, 2024
